# Characterizing the immune response to myocardial infarction in pigs

**DOI:** 10.1007/s00395-024-01036-2

**Published:** 2024-03-15

**Authors:** Florian Schnitter, Franziska Stangl, Elisabeth Noeske, Maya Bille, Anja Stadtmüller, Niklas Vogt, Florian Sicklinger, Florian Leuschner, Anna Frey, Laura Schreiber, Stefan Frantz, Niklas Beyersdorf, Gustavo Ramos, Nadine Gladow, Ulrich Hofmann

**Affiliations:** 1 Department of Internal Medicine I, University Hospital Würzburg, Würzburg, Germany; 2 Comprehensive Heart Failure Center, University Hospital Würzburg, Würzburg, Germany; 3 Comprehensive Heart Failure Center, Department of Cardiovascular Imaging, University Hospital Würzburg, Würzburg, Germany; 4 Institute of Pathology, University of Würzburg, Würzburg, Germany; 5 Institute for Virology and Immunobiology, University of Würzburg, Würzburg, Germany; 6 Department of Internal Medicine III, University Hospital Heidelberg, Heidelberg, Germany; 7 DZHK (German Centre for Cardiovascular Research), Partner Site Heidelberg, Heidelberg, Germany

**Keywords:** Myocardial infarction, Pig, Swine, Heart, Leukocytes, Immunophenotyping

## Abstract

Though myocardial infarction (MI) in pigs is a well-established translational large animal model, it has not yet been widely used for immunotherapy studies, and a comprehensive description of the immune response to MI in this species is lacking. We induced MI in Landrace pigs by balloon occlusion of the left anterior descending artery over 90 min. Within 14 days, the necrotic myocardium was progressively replaced by scar tissue with involvement of myofibroblasts. We characterized the immune response in the heart ex vivo by (immuno)histology, flow cytometry, and RNA sequencing of myocardial tissue on days 3, 7, and 14 after MI. Besides a clear predominance of myeloid cells among heart-infiltrating leukocytes, we detected activated T cells and an increasing proportion of CD4^+^ Foxp3^+^ regulatory T cells (T_reg_), especially in the infarct core—findings that closely mirror what has been observed in mice and humans after MI. Transcriptome data indicated inflammatory activity that was persistent but markedly changing in character over time and linked to extracellular matrix biology. Analysis of lymphocytes in heart-draining lymph nodes revealed significantly higher proliferation rates of T helper cell subsets, including T_reg_ on day 7 after MI, compared to sham controls. Elevated frequencies of myeloid progenitors in the spleen suggest that it might be a site of emergency myelopoiesis after MI in pigs, as previously shown in mice. We thus provide a first description of the immune response to MI in pigs, and our results can aid future research using the species for preclinical immunotherapy studies.

**Supplementary Information:**

The online version contains supplementary material available at 10.1007/s00395-024-01036-2.

## Introduction

Due to its genetic, anatomical, and physiological similarities to our species, the domestic pig (*Sus scrofa domesticus*) is increasingly recognized as a valuable organism for modeling a wide variety of human diseases [49]. In cardiovascular research, pigs are already a well-established translational large animal model for studying myocardial infarction (MI) by applying open- or closed-chest techniques to induce regional ischemia and generate myocardial injury with or without subsequent reperfusion [25]. The type, extent, and pattern of spatial distribution and the temporal sequence of ischemia–reperfusion in pigs resemble the human clinical situation more closely than many other animal models [19, 33]. Moreover, utilizing pigs allows researchers to employ the same techniques, such as cardiac catheterization or magnetic resonance imaging, and derived key parameters as in examinations of the human heart [57, 64].

During the last decade, the immune response to MI received growing interest. A broad array of immunological and genetic tools, primarily available for mice, has provided us with deep insights into the functional heterogeneity of innate and adaptive immune cell responses to MI in rodent models [80]. However, most of this knowledge is based on findings from inbred young animals kept under specific pathogen-free conditions—a major, often unaddressed caveat in immunological research. Using conventionally raised pigs for cardioimmunological studies would help overcome some of the limitations inherent in laboratory mice and holds promise for bridging the translational gap. The fundamentally varied rearing and housing conditions of regular farm pigs together with several routine vaccinations result in animals with a much more antigen-experienced and genetically diverse adaptive immune system, even at young ages. Recent comparative genomic and transcriptomic studies have underlined the closer immunological proximity of pigs to humans [12] and strengthened the significance of porcine models for immunological research in general [53]. This applies to both the innate and adaptive branches of the immune system [18, 32]. Hence, to further evaluate the translational potential of immunotherapeutic approaches in the context of MI, the pig appears to be a particularly useful model for indispensable large animal studies. Yet, only a few immunotherapeutic strategies developed in mice have been validated in pigs [29, 61, 76]. Moreover, there are scant reports on leukocytes in the porcine heart, and the immune response to ischemic cardiac injury, despite being the actual target of such therapeutic interventions, has not yet been systematically studied [27, 74].

Based on these encouraging findings and outstanding knowledge gaps, our main objective in this study was to describe suitable approaches to investigate the immune response to experimental MI in pigs, with a focus on cellular protagonists and their spatiotemporal dynamics during the early healing process. We chose a closed-chest model of MI induced by transient balloon occlusion of the left anterior descending artery (LAD) in Landrace pigs. In contrast to open-chest models, which typically involve surgical coronary ligation, we minimized procedure-related tissue trauma and subsequent immune alterations. Furthermore, reperfusion of infarcted tissue after 90 min brings the model close to clinical reality in human MI patients undergoing interventional therapy. Our analyses were performed on tissues from days 3, 7, and 14 after MI to characterize immunological phenomena during the early inflammatory phase, the proliferative or transitional phase toward healing of the injured myocardium, and the beginning resolution phase, characterized by scar maturation in the infarct region. The macroscopic aspect of the tissue, the histologic appearance, and the observed degree of fibrosis supported our choice of these points in time after MI. Using antigenic markers reported in the literature for the identification of myeloid and lymphoid immune cell populations in porcine extracardiac tissues, we describe here their first application to immunofluorescence microscopy and flow cytometry of tissue samples from the infarcted pig heart. In addition, we used flow cytometry to examine heart-draining lymph nodes and the spleen as secondary lymphoid organs likely involved in the post-MI immune response. Moreover, we present regional RNA-seq transcriptome data to characterize the prevalent milieu in affected cardiac tissue as a resource for further research. All these technical advances will also aid to complement and validate future single-cell transcriptomic analyses of leukocytes. We hope that our work will help us move forward on the translational path of cardioimmunology by facilitating therapeutic studies in pigs.

## Methods

### Pig MI model

**Fig. 1:**
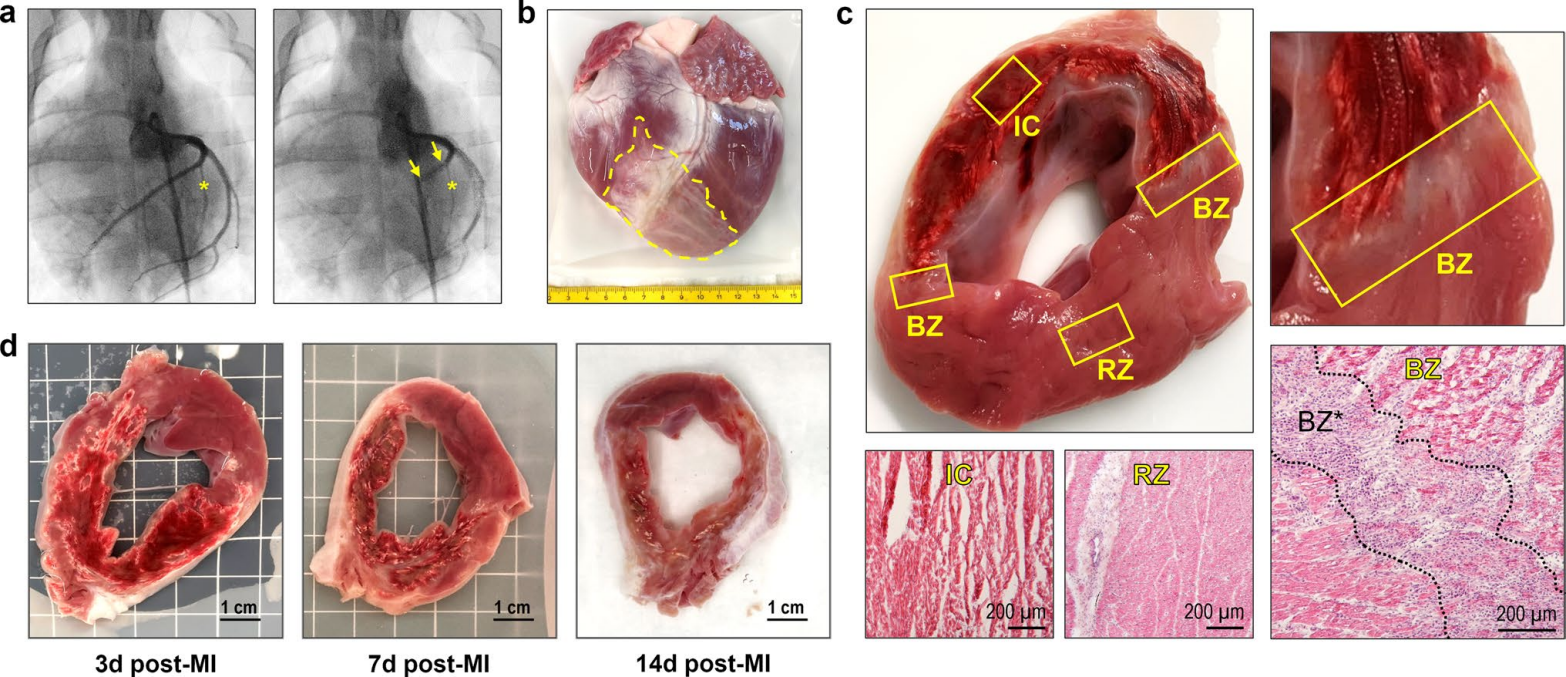
Pig MI model, macroscopic features of the infarcted porcine heart and cardiac tissue sampling. **a** Coronary angiography before (left) and during (right) MI induction. Interventional LAD occlusion was performed by temporary (90 min) inflation of a coronary balloon (yellow arrows) distal from the first diagonal branch (yellow asterisk). **b** Explanted pig heart at 14 days post-MI. The pale infarct/scar region (yellow dashed line) can be clearly identified. **c** LV cross-section at 3 days post-MI with sampled regions and corresponding hematoxylin–eosin (H.E.)-stained microscopic overview images. Tissue samples were routinely collected from the infarct core (IC), both border zones (BZ), and the remote zone (RZ). Note the extensive hemorrhage in the infarct area and the localization of the border zones at its two edges, which can be more precisely delineated microscopically (BZ*). **d** Exemplary LV cross-sections at the indicated points in time (d = days) post-MI

12–18-Week-old healthy female German Landrace pigs with an average body weight (BW) of 40.4 ± 8.7 kg (source: Gerd Heinrichs, Heinsberg, Germany) were sedated by intramuscular injection of azaperone (2–4 mg/kg BW) plus atropine (0.5 mg) followed by ketamine (15–25 mg/kg BW). Subsequently, two endovenous catheters were placed in the auricular veins. Lidocaine was sprayed on the glottis region and after intravenous bolus administration of propofol (2 mg/kg BW), endotracheal intubation was performed. The pigs were placed on an operating table covered with an electric heating mat. Monitoring of vital parameters (heart rate, blood pressure, peripheral blood oxygen saturation, three-lead ECG, and body temperature) was established. Then, general anesthesia was induced and maintained by intravenous infusion of propofol (5 mg/kg BW/h) and fentanyl (30–50 µg/kg BW/h). The animals were taken on mechanical ventilation (pressure control, fraction of inspired oxygen 0.6) with capnometry monitoring. Drug treatment was complemented by continuous intravenous infusion of amiodarone (2 mg/kg BW/h) for arrhythmia prophylaxis and of balanced electrolyte solution for euvolemia. After ultrasound-guided puncture of the femoral artery a 6 French sheath was inserted in the vessel. Following intravenous bolus administration of heparin (225 international units/kg BW), a hockey stick guiding catheter was introduced and baseline left coronary angiography was performed. Next, a coronary angioplasty balloon catheter was advanced over a coronary guide wire into the proximal to mid LAD and the balloon was inflated for complete vessel occlusion distal of the first diagonal branch, as angiographically verified (Fig. 1a). During the 90-min coronary occlusion, balloon pressure was maintained at 6 bar and vital parameters were closely monitored. Malignant ventricular arrhythmia, if detected in the ECG recording, was immediately treated by biphasic defibrillation at 200 J. Full cardiac resuscitation, including chest compressions and intravenous adrenaline bolus (1 mg) injection, when indicated, was provided for a maximum of 10 min. If these measures failed to restore a stable spontaneous circulation, the animals were euthanized by intravenous bolus injection of sodium pentobarbital (150 mg/kg BW). At the end of the occlusion period, coronary angiography was performed directly before and after balloon deflation to ensure successful reperfusion of the LAD. Animals assigned to the sham group underwent an identical procedure, but without the insertion of an angioplasty balloon catheter via the guidewire and therefore without coronary occlusion. Eventually, the coronary catheters as well as the vascular sheath were removed and the puncture site was treated with a closure system (Exoseal; Cordis, Miami Lakes, FL, USA). General anesthesia was terminated, and meloxicam (0.4 mg/kg BW) was administered intramuscularly as post-interventional pain medication. The extubated pigs were finally placed in a recovery box where they were closely monitored until they regained full consciousness. Intramuscular injection of meloxicam was repeated by default on the following day.

3, 7 or 14 days after the procedure, pigs were sedated by intramuscular injection of midazolam (0.3–0.5 mg/kg BW) and ketamine (15–25 mg/kg BW). One auricular vein catheter was placed for blood collection and heparin bolus (225 international units/kg BW) administration. Finally, animals were euthanized by intravenous bolus injection of sodium pentobarbital (150 mg/kg BW) and sectioning was started immediately.

A total of 19 animals underwent temporary balloon occlusion of the LAD (MI procedure). Of these, 6 animals died (3 during the MI procedure due to malignant arrhythmia and 3 within 24 h after the procedure by sudden cardiac death). The surviving animals were sacrificed as described above after 3 days (*n* = 3), 7 days (*n* = 5), and 14 days (*n* = 5). In addition, 6 animals underwent the sham procedure in which only the coronary balloon occlusion was omitted. No animal died in the sham group. These animals were also sacrificed after 3 days (*n* = 4), 7 days (*n* = 1), and 14 days (*n* = 1). Unless otherwise stated in the figure legends, we pooled all sham animals for further analyses. Considering the 3R principles, we did not form separate sham groups for each point in time, as these animals experienced only minor tissue injury related to the femoral artery puncture (in contrast to animals subjected to significantly more invasive open-chest procedures).

### Heart processing and sampling strategy

Explanted pig hearts were thoroughly washed in ice-cold 0.9% saline. Additionally, to minimize intravascular contaminants, both coronary ostia were cannulated, and the downstream vessels were manually perfused with at least 150 ml saline on each side until visually free of blood. The left ventricle (LV) was isolated and cut into transverse slices of equal thickness (approximately 8–10 mm). Representative tissue samples were then obtained from at least two locations within the infarct core, the adjacent border zone, and the non-infarcted remote zone—all identified solely by visual macroscopic inspection of the unstained tissue (Fig. 1c). The infarcted area was distinguished from viable myocardium based on differences in color and texture. Related specimens for flow cytometry and RNA isolation were pooled in equal proportions before further processing.

### Cryoembedding and -sectioning of cardiac tissue

Cardiac tissue samples were cryoembedded in optimal cutting temperature compound (Tissue-Tek; Sakura Finetek Europe, Alphen aan den Rijn, The Netherlands) on dry ice using isopentane and preserved at  – 80 °C. Later, tissue blocks were frozen sectioned at 15 µm (for H.E. and PSR staining) or at 7 µm thickness (for immunofluorescence staining) with a cryostat microtome (CM1850; Leica Biosystems, Wetzlar, Germany), placed on glass slides, and either directly stained or stored at  – 20 °C for a maximum of a few days until staining.

### H.E. (hematoxylin–eosin) staining of porcine cardiac tissue

Thawed cryosections were fixed for 30 min in 4% PFA solution. Next, samples were stained with Mayer’s 0.1% hematoxylin solution (Morphisto, Offenbach, Germany) for 10 min, then blued in running tap water for 10 min, and counterstained with a 0.3% solution of eosin Y (Morphisto) for 5 min. After washing with distilled water (dH_2_O), dehydration through an ascending series of ethanol, and clearing in xylene as well as xylene substitute (ROTI-Histol; Carl Roth, Karlsruhe, Germany), samples were mounted under coverslips with rapid mounting medium (Entellan; Merck, Darmstadt, Germany) for subsequent examination under the light microscope (Axioskop 2 plus, Carl Zeiss Microscopy, Jena, Germany).

### H.E.-stained human cardiac tissue

Digital light microscopic images of formalin-fixed, paraffin-embedded, and H.E.-stained tissue Sects. (3–5 µm thickness) from human left-ventricular myocardium were provided by the Institute of Pathology, Würzburg, Germany. Tissue samples had been obtained during autopsy of patients deceased at different points in time (until approximately 14 days) after MI.

### Picrosirius red (PSR) staining of porcine cardiac tissue

Thawed cryosections were fixed for 30 min in 4% PFA solution. Then, samples were stained with 0.01% PSR solution (Morphisto, Offenbach, Germany) for 60 min. After washing with dH_2_O, dehydration through an ascending series of ethanol, and clearing in xylene as well as xylene substitute (ROTI-Histol; Carl Roth, Karlsruhe, Germany), samples were mounted under coverslips with rapid mounting medium (Entellan; Merck, Darmstadt, Germany).

Subsequent examination under the fluorescence microscope (DMi8; Leica Microsystems, Wetzlar, Germany) allowed a clear distinction between PSR-positive (i.e., collagen) and negative areas based on their different fluorescence characteristics [75]. Using a grid scheme, representative images were taken from the infarct core (9 images per section), the more homogeneous remote zone and from sham hearts (5 images per section). The PSR-positive area and the total tissue area were determined planimetrically from the digital images using Photoshop CS5 (Adobe, San José, CA, USA). Endo-/epicardium, larger vessels, and artifacts were excluded. Eventually, the average collagen content of each section was calculated as the mean ratio (%) of PSR-positive (collagen) area to total tissue area of all related images.

### Immunofluorescence staining of cardiac tissue

Thawed cryosections were fixed in 4% PFA for 30 min and washed in Dulbecco’s phosphate-buffered saline (DPBS; 3 × 5 min each). After permeabilization with 0.02% Triton X-100 (Sigma-Aldrich, St. Louis, MO, USA) for 10 min, washing steps were repeated. Depending on the species of origin of the secondary antibodies, samples were then blocked with either 3% mouse (Biozol, Eching, Germany), 3% goat or 5% rat serum (Vector Laboratories, Newark, CA, USA) for 60 min. Further incubation with the respective primary antibodies (Online Resource 1: Table 1; panels 5–8) diluted in the blocking solution was performed in a humid chamber, either overnight at 4 °C or for 2 h at room temperature. Slides were then washed in DPBS (3 × 5 min each) and stained with the appropriate secondary antibodies (Online Resource 1: Table 2; panels 5, 7, 8) for 120 min at room temperature. Washing steps were repeated. In some cases, several such incubations with primary and the respective secondary antibodies had to be performed consecutively to avoid cross-reactions. Finally, nuclei were stained with 4′,6-diamidino-2-phenylindole (DAPI, 1:5000) for 10 min at room temperature. After washing, samples were mounted under coverslips with aqueous mounting medium (Mowiol 4–88; Kuraray Europe, Hattersheim, Germany) and then visualized under the fluorescence microscope (DMi8; Leica Microsystems, Wetzlar, Germany).

For quantification of immunofluorescence staining, representative images were obtained from the infarct core, border zone (3–5 images per section), remote zone, and sham hearts (2–3 images per section). The area (co)staining for the respective markers and the total tissue area were determined planimetrically from the digital images using the Fiji v2.15.0 distribution of ImageJ2 [63]. Endo-/epicardium, larger vessels, and artifacts were excluded. Eventually, the relative abundance of cells of interest in each region was approximated as the mean ratio (%) of (co)stained area to total tissue area in all related images.

### RNA isolation from cardiac tissue

Small pieces of cardiac tissue were collected in RNA stabilization solution (RNAlater; Thermo Fisher Scientific, Waltham, MA, USA) and incubated overnight at 4 °C. Then, pieces were taken out and shock frosted in liquid nitrogen for further preservation at  – 80 °C. For RNA isolation, tissue pieces were thawed and mechanically homogenized with a TissueRuptor II (Qiagen, Hilden, Germany) in buffer RLT from the RNeasy mini kit (Qiagen, Hilden, Germany). Samples were further processed with the kit following the manufacturer’s instructions. Finally, RNA concentration and purity (260/280 ratio) in the column eluates were determined using a spectrophotometer (NanoDrop 2000c; Thermo Fisher Scientific, Waltham, MA, USA), and the samples were stored at  – 20 °C.

### Bulk RNA sequencing (RNA-seq) from cardiac tissue

RNA quality in the thawed samples was checked using a 2100 Bioanalyzer with the RNA 6000 Nano kit (Agilent Technologies, Santa Clara, CA, USA). The RNA integrity number (RIN) for all samples was ≥ 7.0. DNA libraries suitable for sequencing were prepared from 400 ng of total RNA with oligo-dT capture beads for poly-A-mRNA enrichment using the TruSeq Stranded mRNA Library Preparation Kit (Illumina, San Diego, CA, USA) according to the manufacturer’s instructions (½ volume). After 14 cycles of PCR amplification, the size distribution of the barcoded DNA libraries was estimated ~ 305 bp by electrophoresis on Agilent DNA 1000 Bioanalyzer microfluidic chips. Sequencing of pooled libraries, spiked with 1% PhiX control library, was performed at 35 million reads/sample in single-end mode with 100 nt read length on the NextSeq 2000 platform (Illumina) using a P3 sequencing kit. Demultiplexed FASTQ files were generated with bcl2fastq2 v2.20.0.422 (Illumina). To assure high sequence quality, Illumina reads were quality- and adapter-trimmed via Cutadapt [50] v2.5 using a cut-off Phred score of 20 in NextSeq mode, and reads without any remaining bases were discarded (command line parameters: –nextseq-trim = 20 -m 1 -a AGATCGGAAGAGCACACGTCTGAACTCCAGTCAC). Processed reads were subsequently mapped to the Pig genome (Sus scrofa GCF_000003025.6_Sscrofa11.1) using STAR v2.7.2b with default parameters [15]. Read counts on exon level summarized for each gene were generated using featureCounts v1.6.4 from the Subread package [41]. Multi-mapping and multi-overlapping reads were counted strand-specific and reversely stranded with a fractional count for each alignment and overlapping feature (command line parameters: -s 2 -t exon -M -O –fraction). The count output was utilized to identify differentially expressed genes using DESeq2 [47] v1.24.0. Read counts were normalized by DESeq2 and fold-change shrinkage was applied by setting the parameter “betaPrior = TRUE”. Differential expression of genes was assumed at an adjusted p value (padj) after Benjamini–Hochberg correction < 0.05 and |log_2_ fold change|≥ 1.

The 15,677 differentially expressed genes in MI samples (infarct core) vs. sham were loaded into GSEA (gene set enrichment analysis) software for appropriate analysis [67], using the human Molecular Signatures Database (MSigDB) hallmark gene sets collection (h.all.v7.5.1.symbols.gmt) [42]. GSEA performed 1000 permutations. The maximum and minimum sizes for gene sets were 500 and 15, respectively. Gene sets with a false discovery rate (FDR) < 25% were considered significantly enriched. From selected hallmark gene sets, the top differentially expressed genes with log_2_ fold change > 2 in MI samples (infarct core) vs. sham were mapped to the STRING (Search Tool for the Retrieval of Interacting Genes/Proteins) database v11.5 [70] (interaction score threshold 0.7, FDR < 0.1%) to identify potential protein–protein interaction networks. Within these networks, functional clusters were delineated based on Gene Ontology (GO) terms.

### Flow cytometry of immune cells enriched from pig hearts

For flow cytometry, finely minced cardiac tissue samples were digested in Hanks’ balanced salt solution (HBSS) with 600 units/ml Collagenase Type 2 (Worthington Biochemical, Lakewood, NJ, USA) and 60 units/ml DNAse I (Sigma-Aldrich, St. Louis, MO, USA) for 30 min at 37 °C. The resulting suspension was sequentially filtered through 70 µm and 40 µm cell strainers (incompletely digested tissue was triturated) and washed in cold HBSS^−/−^ (without Ca^2+^ and Mg^2+^) supplemented with 0.1% bovine serum albumin. After red blood cell lysis with ammonium-chloride-potassium (ACK) buffer, staining with a fixable viability dye (Zombie Aqua; BioLegend, San Diego, CA, USA) at a final dilution of 1:200 in DPBS^−/−^/EDTA (without Ca^2+^/Mg^2+^, containing 2 mM ethylene diamine tetra-acetic acid) was performed for 15 min at room temperature. Cells were then washed in cold flow cytometry buffer (DPBS^−/−^/EDTA supplemented with 2% fetal calf serum) and blocked with pig serum (own production; 10% in DPBS^−/−^/EDTA) for 15 min at 4 °C. Next, anti-CD45-FITC antibody (Clone K252.1E4; Biorad, Hercules, CA, USA) was added for a final concentration of 1:50 and samples were incubated for 30 min at 4 °C. CD45 serves as a canonical marker for leukocytes in mice, pigs, and humans. In our protocol, we routinely combined enzymatic tissue digestion with subsequent enrichment of CD45^+^ cells by magnetic activated cell sorting (MACS) to achieve sufficient leukocyte yields, including from the non-infarcted myocardium. Therefore, after washing of the cells, their incubation with anti-FITC MicroBeads (Miltenyi Biotec, Bergisch Gladbach, Germany; final dilution 1:10) and MACS enrichment of CD45^+^ cells over LS Columns (Miltenyi Biotec) were performed according to the manufacturer’s instructions. Pelleted eluates were resuspended in the respective primary antibody master mix (Online Resource 1: Table 1; panels 1, 2) and stained for 30 min at 4 °C. After washing, samples intended for the analysis of myeloid cell surface markers were incubated with the relevant secondary antibodies (Online Resource 1: Table 2; panel 1) for 30 min at 4 °C. The samples designated for the analysis of lymphoid cell markers were fixed and permeabilized using the eBioscience Foxp3/Transcription Factor Staining Buffer Set (Thermo Fisher Scientific, Waltham, MA, USA) per the manufacturer’s instructions to enable subsequent intracellular staining of CD79α and Foxp3 for 45 min at room temperature. Eventually, cells were washed, resuspended in flow cytometry buffer, and measured with the Attune NxT Flow Cytometer (Thermo Fisher Scientific, Waltham, MA, USA). FlowJo v10.0.8 (FlowJo, Ashland, OR, USA) was used for further data analysis.

### Flow cytometric immunophenotyping gating strategy

From at least 10,000 events per sample recorded on the flow cytometer, singlets were selected using FSC-A vs. FSC-H. Within viable (Zombie Aqua^−^) single cells that stained positive for CD45 (i.e., leukocytes), various immune cell subpopulations were then delineated as follows:

Among myeloid immune cells expressing the pan-myeloid marker CD172α, sequential gating (Fig. 10a) based on selected surface antigen markers [17, 56] identified mature macrophages as CD203α^+^. These cells also exhibited markedly increased size and granularity in the forward scatter/side scatter (FSC/SSC) plot compared to the CD203α^−^ cells and showed variable expression of CD14, as detected by a cross-reactive anti-human antibody (TÜK4). Neutrophils and eosinophils, in turn, were characterized by expression of the SWC8 antigen and by their SSC^hi^ scatter profile. They also stained negative for CD163, a molecule restricted to the monocyte/macrophage lineage.

Lymphocytes were primarily distinguished from other leukocytes by their SSC^lo^ FSC^int^ scatter properties. Within this population, T cells were characterized by their surface expression of CD3, whereas B cells were delineated as CD3^−^ CD79α^+^. Among double-negative (CD3^−^ CD79α^−^) cells, NK cells could be detected by their CD8α expression. Further sequential gating of CD3^+^ T lymphocytes (Fig. 11a) using a selection of antigen markers [20, 32] allowed us to discriminate between (1) CD4^+^ CD8α^−^ T cells, resembling naïve T helper (T_H_) cells; (2) CD4^+^ CD8α^+^ T cells, resembling activated T_H_ and memory T cells; (3) CD4^−^ CD8α^+^ T cells, which include activated γδ, cytotoxic (T_cyt_), and NK T cells; and (4) CD4^−^ CD8α^−^ (double-negative) T cells, most closely resembling naïve γδ T cells. Eventually, intracellular staining of the transcription factor Foxp3 enabled us to differentiate *bona fide* CD4^+^ Foxp3^+^ regulatory T cells (T_reg_) from CD4^+^ Foxp3^−^ conventional T helper (T_conv_) cells. In addition, for analysis of T cells from mediastinal lymph nodes, CD8β served as a specific marker for T_cyt_ and CD27 as a surface molecule to differentiate between memory T-cell subsets. For pig CD8β^−^ T cells, CD8α could then be used as activation/differentiation marker [32].

Finally, based on reports regarding hematopoietic cells in pigs [21, 55, 68], we defined stem and progenitor cells as CD117 (c-kit)^+^ but lacking the lymphoid lineage markers CD3 and CD21^a^ (a pan-B cell marker used as an alternative to intracellular staining of CD79α [66]). In turn, expression of the pan-myeloid surface marker CD172α follows appropriate lineage commitment and maturation. For flow cytometry, there is still a lack of commercially available antibodies against porcine CD34, a surface antigen commonly used as a canonical marker for hematopoietic stem and progenitor cells in other species [83].

### Identification of heart-draining mediastinal lymph nodes

In one pig euthanized on day 14 post-MI, thoracotomy was performed, and 2 ml toluidine blue dye were injected directly into the infarcted LV wall. After 5 min, heart-draining lymph nodes could be localized by their dye uptake, as seen on pathological examination of the mediastinal lymphatic vasculature.

### Processing and cryopreservation of extracardiac tissue/cells

Spleen tissue as well as mediastinal lymph nodes were cut into small pieces and rubbed through a 70 μm cell strainer in cold DPBS^−/−^/EDTA. Each cell suspension was then poured over a 40 μm cell strainer. After centrifugation (and red blood cell lysis with ACK buffer for spleen cells), cell numbers were determined with an automated counter (TC20; Biorad, Hercules, CA, USA). Cells were immersed in cryomedium (50% RPMI-1640 medium/40% fetal calf serum/10% dimethyl sulfoxide) at a final concentration of 1 × 10^7^ cells/1.5 ml and preserved at  – 80 °C.

### Flow cytometry of extracardiac cells

Cryopreserved cells were thawed and washed in cold DPBS^−/−^/EDTA. Next, staining with a fixable viability dye (Zombie Aqua; BioLegend, San Diego, CA, USA) at a final dilution of 1:200 in DPBS^−/−^/EDTA was performed for 15 min at room temperature. Cells were then washed in cold flow cytometry buffer and blocked with 10% pig serum (own production) for 15 min at 4 °C. Then, the respective double-concentrated primary antibody master mix (Online Resource 1: Table 1; panels 3, 4) was added in a volume ratio of 1:1 and samples were stained for 30 min at 4 °C. After washing, cells were fixed and permeabilized using the eBioscience Foxp3/Transcription Factor Staining Buffer Set (Thermo Fisher Scientific, Waltham, MA, USA) per the manufacturer’s instructions to enable subsequent intracellular staining (45 min at room temperature) of Foxp3 and/or the cell proliferation marker Ki67. Flow cytometric measurement and subsequent data analysis, including gating, were performed as outlined above.

### Data analysis

Statistical analysis and data visualization were performed using GraphPad Prism v9.4.0 (GraphPad Software, San Diego, CA, USA). Values are presented as mean ± SD if not otherwise indicated. Sample sizes for each group and statistical tests applied are specified in the figure legends. Shapiro–Wilk test was used to check for normal distribution. A *p* value < 0.05 was considered statistically significant.

## Results

### Macroscopic and microscopic characteristics of the infarcted porcine myocardium

Explanted hearts from pigs that underwent 90 min of LAD balloon occlusion with subsequent reperfusion (Fig. 1a) showed extensive, macroscopically discernable infarcts (Fig. 1b). In cross-sections, a consistently large proportion of the anteroseptal to apical left ventricle (LV) was affected by mostly transmural MI (Fig. 1c, d). At 3 days after ischemia–reperfusion, all infarcted areas exhibited widespread hemorrhage, which was still evident on day 7 when these areas had changed color, indicating heme degradation. At 14 days, infarcts mainly consisted of solid, pale connective tissue.

**Fig. 2:**
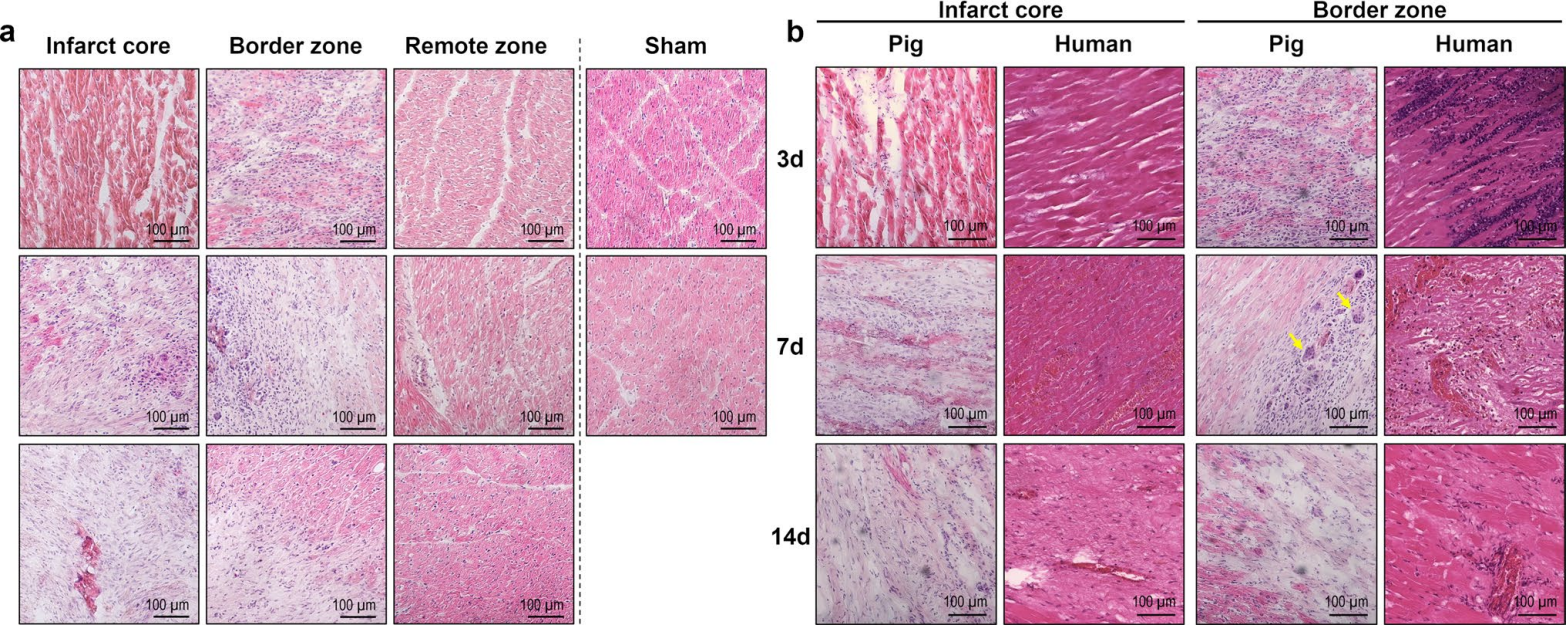
Hematoxylin–eosin (H.E.) staining of porcine and human myocardial tissue. **a** Representative microscopic images from H.E.-stained cryosections of porcine myocardial tissue collected from the different regions at the indicated points in time after MI or sham procedure (d = days). Hypereosinophilia of the necrotic myocardium can be noticed in the infarct core on day 3 post-MI. **b** Comparison of porcine and human H.E.-stained tissue sections from the infarct core and the border zone. Porcine myocardial tissue was cryoembedded on the indicated days post-MI. Autopsy samples from the hearts of human patients who had died at a comparable point in time after MI were formalin-fixed and paraffin-embedded. Multinucleated giant cells are apparent in the border zone of the pig heart 7 days post-MI (yellow arrows)

Histologic assessment of H.E.-stained tissue cryosections (Fig. 2a) from day 3 after MI revealed coagulation necrosis of cardiomyocytes in the infarct core with cell shrinkage, hypereosinophilia, and loss of nuclei and striations. The border zone contained a ribbon-shaped immune cell infiltrate. By comparison, we found 7-day-old infarcts to be much more heterogeneous. The core region exhibited a mixture of granulation tissue with fibroblasts as well as abundant leukocyte infiltrates, primarily focused on remnants of necrotic cardiomyocytes. We noted occasional multinucleated giant cells, mostly adjacent to the border zone. However, there were still areas of necrotic myocardium, apparently unchanged from day 3, with very little immune cell infiltration. After 14 days, the granulation tissue in the infarcted area was mainly fibrous, with regions of dense collagen deposition and neovessel formation. Immune cell accumulation appeared sparse, and leukocytes were centered around residual clusters of necrotic cardiomyocytes. Overall, these findings compare well to observations of autopsy samples from human hearts at similar points in time after MI (Fig. 2b).

**Fig. 3:**
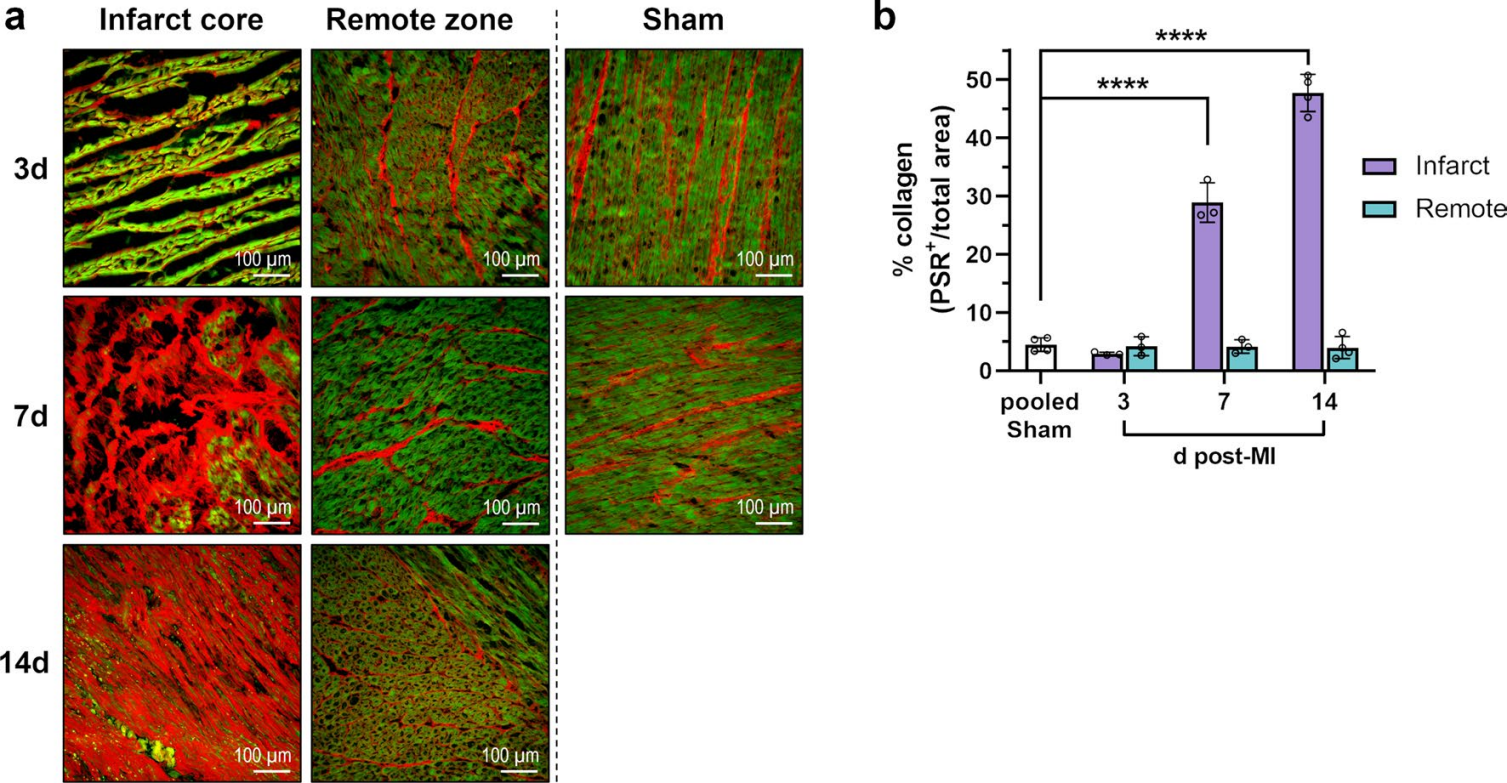
Picrosirius red (PSR) staining of collagen in porcine heart tissue. **a** Representative fluorescence microscopy images from PSR-stained cryosections collected from the infarct core and the border zone at the indicated points in time after MI or sham procedure (d = days; green autofluorescence: cardiomyocytes, red fluorescence: PSR-positive collagen fibers). **b** Myocardial collagen content as determined by fluorescence microscopy of PSR-stained tissue cryosections. Mean ± SD; ****p < 0.0001 vs. pooled sham (two-way ANOVA with Dunnett’s multiple comparisons test)

To examine immune-modulated scar formation during cardiac healing, myocardial collagen content was determined by PSR staining of tissue cryosections (Fig. 3). In non-infarcted myocardium, 4% of total tissue area stained PSR-positive, indicating collagen. No significant change from this baseline was observed in 3-day-old infarcts. In contrast, 7 days after MI the infarcted area had a markedly higher mean collagen content of 29%, though fibers appeared loose and were non-homogeneously distributed. In 14-day-old infarcts, the average collagen content reached 48%, with more compact and organized fiber deposition.

**Fig. 4:**
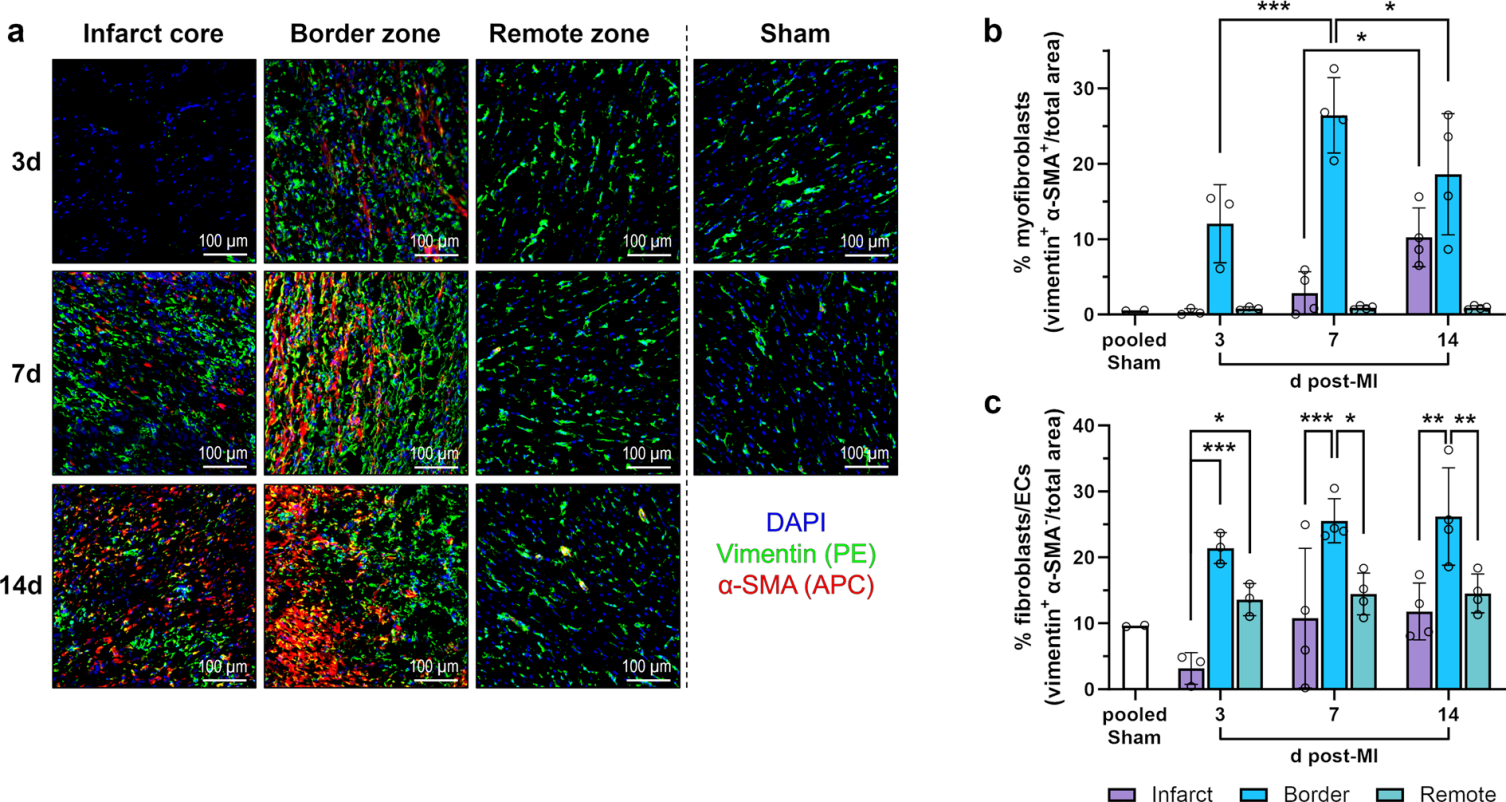
Immunofluorescence staining of (myo)fibroblasts in the porcine myocardium. **a** Representative immunofluorescence microscopy images from cryosections of myocardial tissue collected from the different regions at the indicated points in time after MI or sham procedure (d = days). Cells of mesenchymal origin, such as (myo)fibroblasts and endothelial cells (ECs), were identified by staining for the intermediate filament protein vimentin (green). Among these cells, myofibroblasts characteristically costained for the contractile protein α-smooth muscle actin (α-SMA; red). DAPI (4′,6-diamidino-2-phenylindole) was used for nuclear counterstaining (blue). **b**–**c** Relative abundance of b myofibroblasts or c fibroblasts/ECs as determined by immunofluorescence microscopy of stained tissue cryosections. Mean ± SD; *p < 0.05, **p < 0.01, ***p < 0.001 vs. indicated post-MI samples (two-way ANOVA with Tukey’s multiple comparisons test)

Immunofluorescence microscopy was used to detect vimentin^+^ α-smooth muscle actin (α-SMA)^+^ myofibroblasts [30] (Fig. 4a), which started to accumulate in the border zone on day 3 and reached a peak there on day 7. In the infarct core, their frequency increased significantly from day 7 to day 14 (Fig. 4b). At all points in time after MI, vimentin^+^ α-SMA^−^ cells, i.e., fibroblasts and endothelial cells [14], were most abundant in the border zone. In the infarct core, they were substantially reduced on day 3 compared to the other myocardial regions (Fig. 4c).

Overall, within 14 days after MI, the necrotic myocardium was progressively replaced by a collagenous scar. To investigate the central role of leukocytes during this stage of cardiac healing, we decided to focus our further analyses on this period.

### Identification of leukocytes in cardiac tissue via immunofluorescence microscopy

**Fig. 5:**
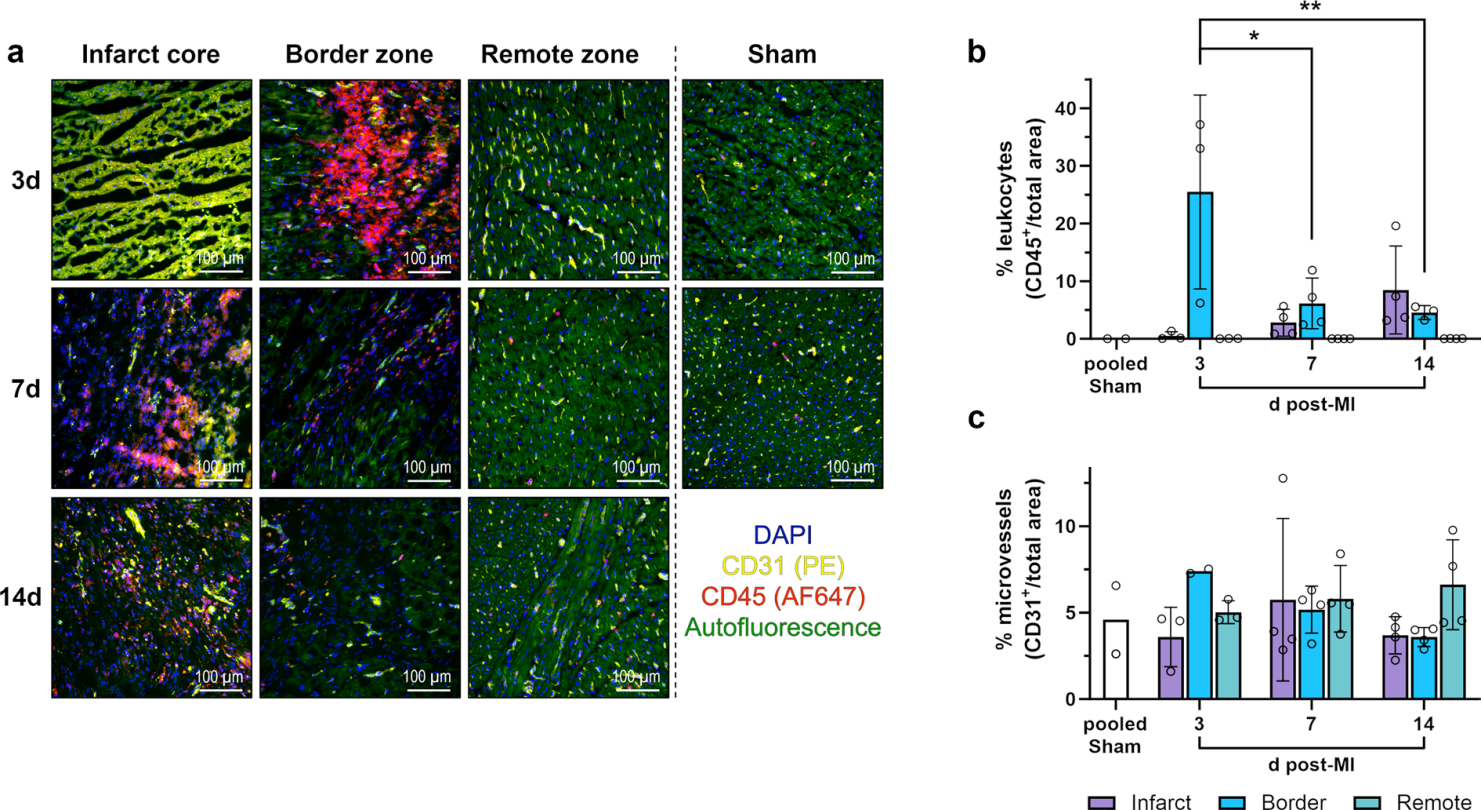
Immunofluorescence staining of leukocytes and microvessels in the porcine myocardium. **a** Representative immunofluorescence microscopy images from cryosections of myocardial tissue collected from the different regions at the indicated points in time after MI or sham procedure (d = days). Leukocytes were identified by staining for the pan-leukocyte marker CD45 (red). Endothelial cells lining vessels stain positive for CD31 (yellow). DAPI (4′,6-diamidino-2-phenylindole) was used for nuclear counterstaining (blue). Necrotic cardiomyocytes exhibit a distinctive yellow-green autofluorescence clearly different from the less intense green autofluorescence of intact cardiomyocytes. **b**–**c** Relative abundance of b leukocytes or c microvessels as determined by immunofluorescence microscopy of stained tissue cryosections. Mean ± SD; *p < 0.05, **p < 0.01 vs. 3d post-MI: border zone (two-way ANOVA with Tukey’s multiple comparisons test)

Immune cells in pig hearts were identified by staining tissue cryosections with antibodies against the pan-leukocyte marker CD45 (Fig. 5a, b). At 3 days post-MI, CD45^+^ cells had densely accumulated in the border zone between necrotic and surviving myocardium. Most leukocytes appeared extravascular as they did not colocalize with CD31^+^ endothelial cells. The infarct core was dominated by necrotic cardiomyocytes showing high autofluorescence. CD45^+^ cells and intact vessels were rare. In 7-day-old infarcts, the leukocyte infiltrate had shifted from the border zone, where most of the dead cardiomyocytes had been cleared up, to the infarct core, where CD45^+^ cells were clustered around necrotic cardiomyocytes. At 14 days after MI, fibrous remodeling of the infarct core was evident and CD45^+^ leukocytes appeared more scattered, but clusters of them persisted around remaining islets of necrotic myocardium. Also, newly formed CD31^+^ capillaries could be recognized in the infarct core and border zone. Yet, the proportion of CD31^+^ endothelial cells [6] lining microvessels did not change significantly across the different points in time and regions (Fig. 5c).

**Fig. 6:**
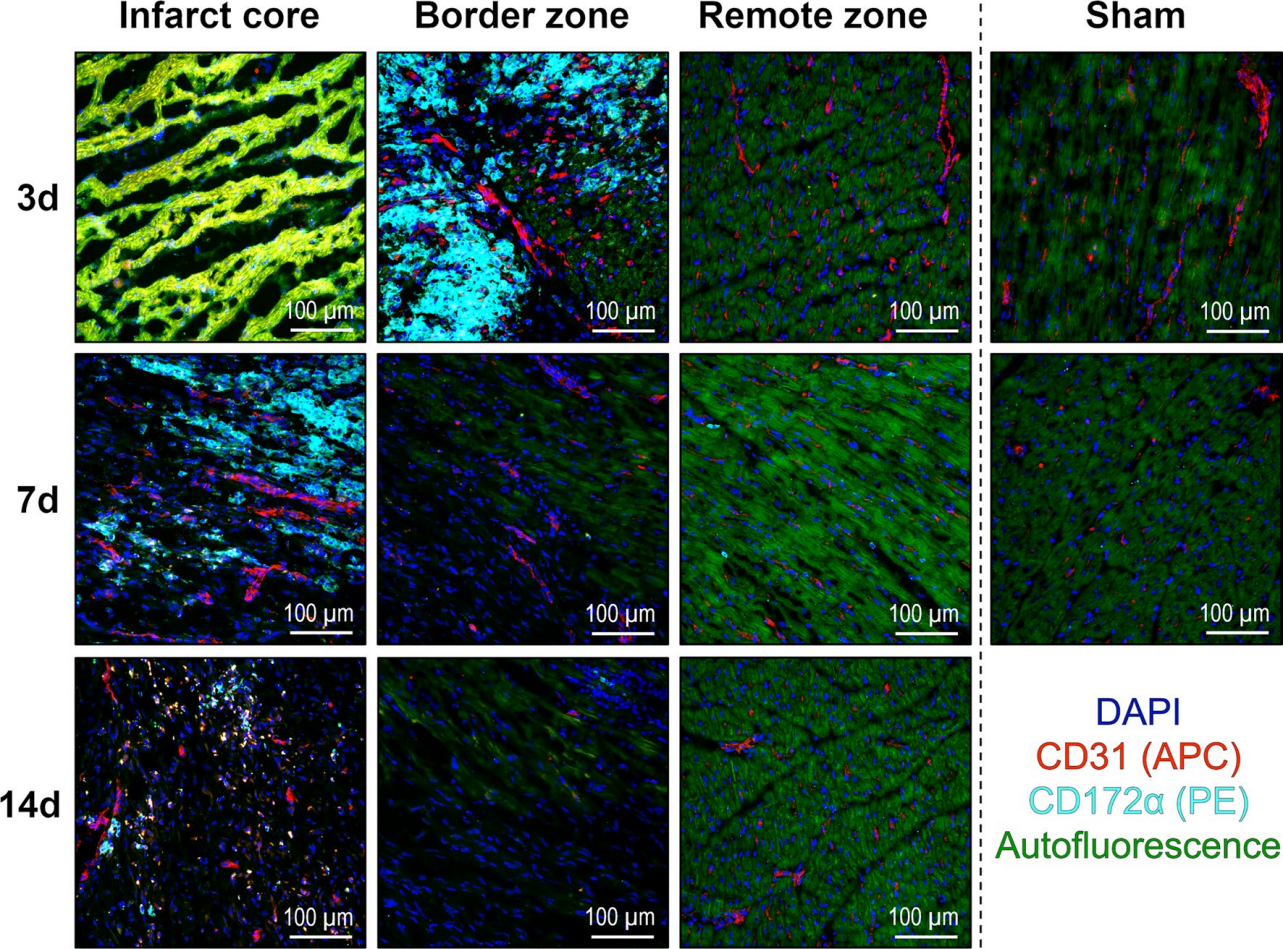
Immunofluorescence staining of myeloid immune cells in the porcine myocardium. Representative immunofluorescence microscopy images from cryosections of myocardial tissue collected from the different regions at the indicated points in time after MI or sham procedure (d = days). Myeloid immune cells were identified by staining for the pan-myeloid cell marker CD172α (cyan). Endothelial cells’ stain positive for CD31 (red). DAPI (4′,6-diamidino-2-phenylindole) was used for nuclear counterstaining (blue). Necrotic cardiomyocytes exhibit a distinctive yellow–green autofluorescence clearly different from the less intense green autofluorescence of intact cardiomyocytes

Additional staining with antibodies against the porcine pan-myeloid cell surface marker CD172α [17] indicated that most infiltrating leukocytes were either monocytes, macrophages, or granulocytes (Fig. 6). At all points in time, we observed no apparent changes in the number or distribution of leukocytes in the remote zone compared with tissue from non-infarcted sham hearts.

### RNA sequencing to characterize the local cardiac tissue milieu after MI

**Fig. 7:**
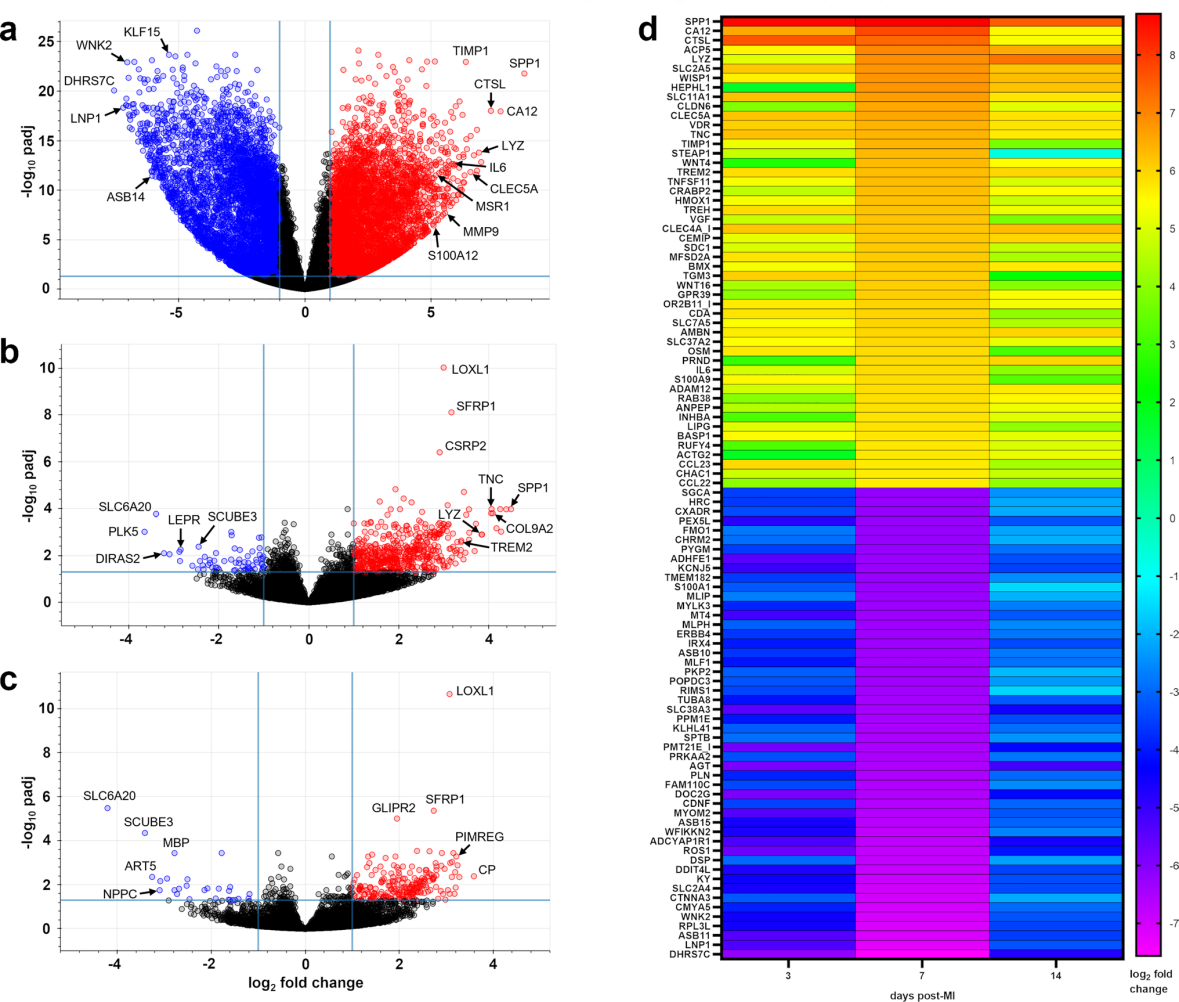
Differential gene expression in infarcted porcine myocardium as determined by bulk-tissue RNA sequencing. **a**–**c** Volcano plots illustrating significantly (|log_2_ fold change|≥ 1.0, adjusted p (padj) ≤ 0.05; corresponding cut-off lines in light blue) up- (red) and downregulated (blue) transcripts in a the infarct core, b the border zone, and c the remote zone vs. pooled sham at 7 days post-MI. d Heatmap representing the most differentially up- and downregulated genes in the infarct core at the indicated points in time post-MI. The color scale shows the log_2_ fold change vs. pooled sham. Transcripts were sorted by log_2_ fold change on day 7 post-MI. padj ≤ 0.05. All calculations performed in DESeq2 (n = 3–4)

**Fig. 8:**
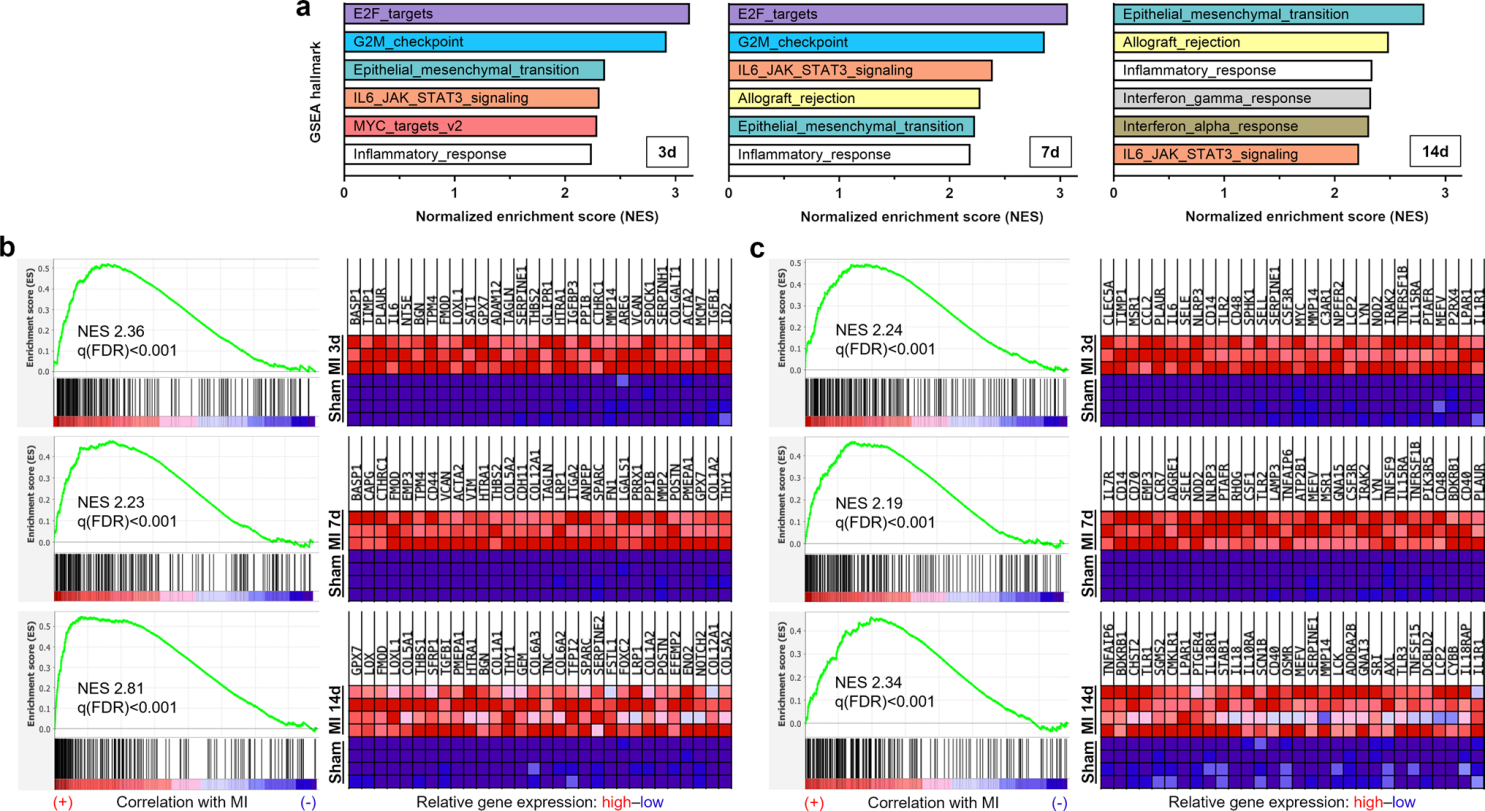
Gene set enrichment analysis (GSEA) of differentially expressed genes in infarcted porcine myocardium as determined by bulk-tissue RNA sequencing. **a** GSEA of differentially expressed genes using the hallmark gene set collection (Molecular Signatures Database—MSigDB). Bar graphs depict the top 6 hallmark gene sets as ranked by their normalized enrichment score (NES), being significantly (q(FDR) < 0.25) enriched in tissue from the infarct core compared to tissue from sham hearts (*n* = 3–4) at the indicated points in time (d = days) post-MI. q(FDR): q value indicating the expected false discovery rate (FDR). **b**–**c** Enrichment plots and corresponding heatmaps for the hallmark gene sets, **b** “epithelial–mesenchymal transition”, and c “inflammatory response” at the indicated points in time after MI. Heatmaps show the relative expression of the top 30 genes in each pathway for the respective post-MI and all sham animals

Complementary to histology, we performed bulk-tissue RNA sequencing of samples from the remote zone, border zone, and infarct core on days 3, 7, and 14 post-MI (Fig. 7; Online Resource 2). Compared to sham, the most differentially expressed genes were found within the infarct core on all days. Therefore, we performed gene set enrichment analysis (GSEA) focused on the infarct core to explore changes in transcriptomic patterns over time (Fig. 8). Top hallmark gene sets on day 3 and 7 were related to proliferation, whereas on day 14 the top gene set was “epithelial–mesenchymal transition” (Fig. 8a). Among top ranked genes within the latter gene set on day 3, cytokines, growth factors, plasminogen regulatory proteins, and metalloproteinases (including *IL6, AREG, MMP14, TIMP1, PLAUR, SERPINE1*) were listed, whereas on day 14, genes linked to activated fibroblasts expressing *POSTN* and collagens were prominent (Fig. 8b). The hallmark gene set "inflammatory response" was among the most enriched gene sets throughout all points in time (Fig. 8a). Genes associated with innate immunity, including *CLEC5A, MSR1, CSF3R, NLRP3, TLR2, CCL2,* and *CD14,* were among the top ranked transcripts on day 3, whereas on day 7 and 14, more genes related to lymphocyte biology like *CCR7, CD70, IL7R, LCK,* and *IL18R* appeared (Fig. 8c). Accordingly, the hallmark gene sets “allograft rejection”, enriched for transcripts related to adaptive immune responses, and “interferon gamma response”, indicating activated T cells, were among the top upregulated gene sets in the infarct core on day 14 post-MI (Fig. 8a).

**Fig. 9:**
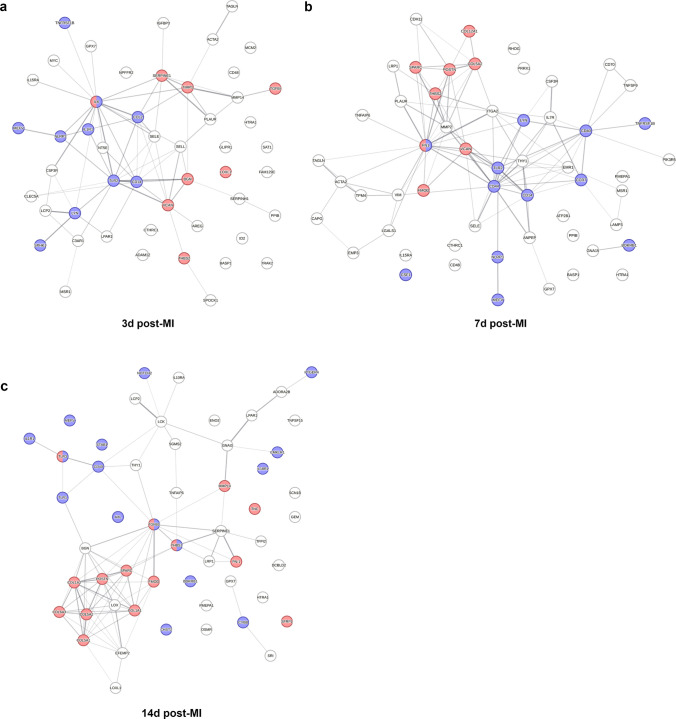
Protein–protein interaction analysis based on bulk-tissue RNA sequencing of infarcted porcine myocardium. **a**–**c** Protein–protein interaction networks generated by mapping the top overexpressed genes from the gene set enrichment analysis hallmarks “epithelial–mesenchymal transition” and “inflammatory response” (log_2_ fold change > 2 in MI samples (infarct core) vs. sham; cf. Figure 8b, c) for a 3 days, b 7 days, or c 14 days post-MI using the STRING (Search Tool for the Retrieval of Interacting Genes/Proteins) database v11.5. Line thickness indicates the strength of data support for protein–protein interactions. Blue dots: proteins associated with the Gene Ontology (GO) functional cluster “inflammatory response” (GO: 0006954), and red dots: proteins associated with the GO cellular component (GOCC) cluster “extracellular matrix” (GOCC: 0031012). All calculations performed online on https://www.string-db.org (*n* = 3–4, interaction score threshold 0.7, false discovery rate < 0.1%)

Furthermore, STRING (Search Tool for the Retrieval of Interacting Genes/Proteins)-based protein–protein interaction analysis (Fig. 9) including the top overexpressed genes of the hallmark sets “inflammatory response” and “epithelial–mesenchymal transition” revealed IL-6, CCL2, TLR2, and CD14 as links between the Gene Ontology (GO) clusters “inflammatory response” and “extracellular matrix biology” on day 3 after MI (Fig. 9a). On day 7, fibronectin (*FN1*), fibromodulin (*FMOD*), and versican (*VCAN*) appeared as additional interconnecting proteins (Fig. 9b). On day 14, the network components changed more profoundly, and thrombospondin (*THBS1*) as well as transforming growth factor-β (*TGFB1*) were identified as links between immunity and extracellular matrix biology (Fig. 9c).

**Fig. 10:**
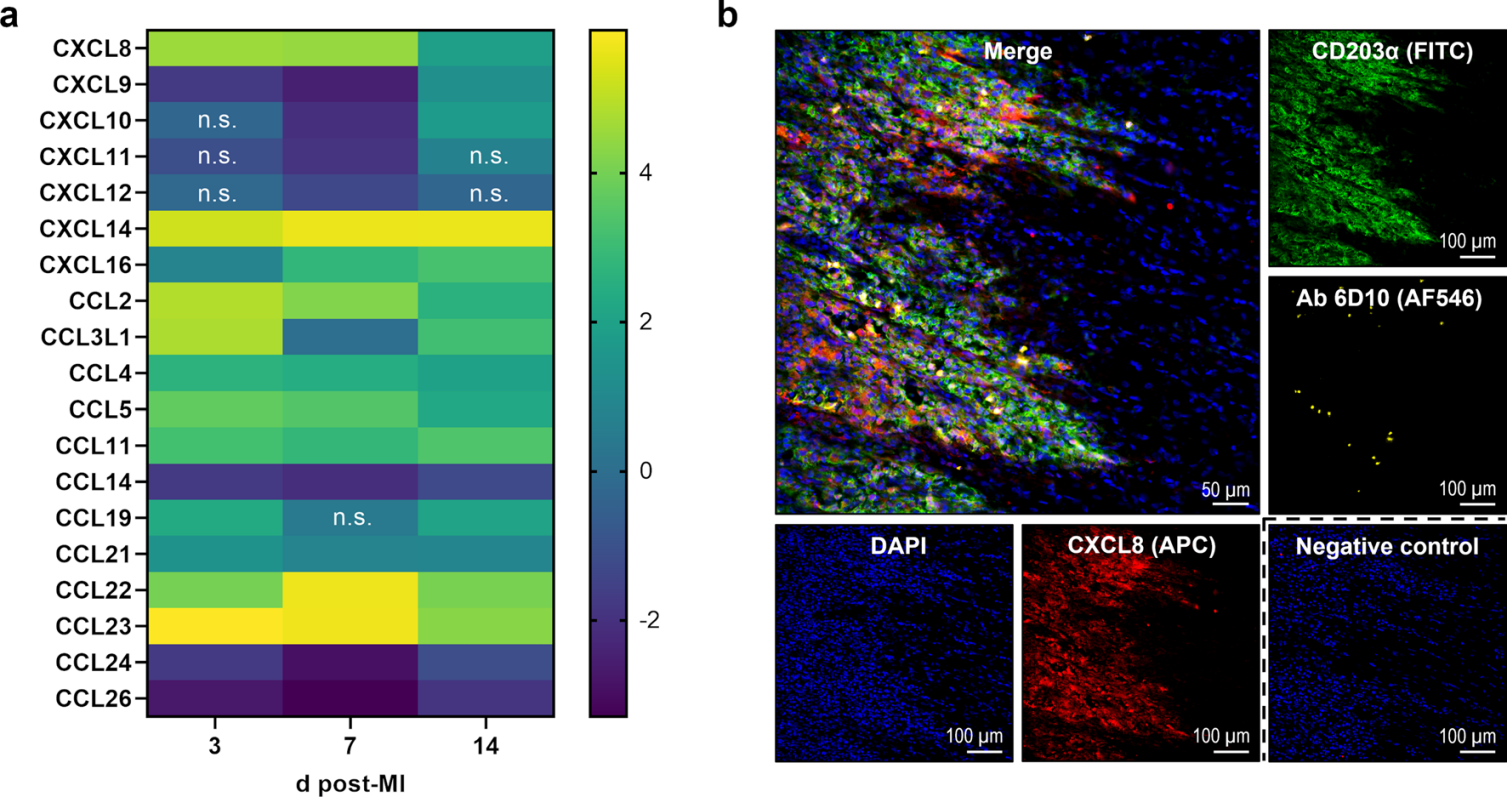
Chemokine expression within the infarcted porcine myocardium. **a** Heat map depicting relative gene expression of CXCL and CCL chemokines determined by bulk RNA sequencing of tissue from the infarct core at the indicated points in time (d = days) post-MI. The color scale shows the log_2_ fold change vs. tissue from sham hearts (pooled) as calculated in DESeq2 (*n* = 3–4). n.s. denotes adjusted *p* > 0.05. **b** Immunofluorescence staining of CXCL8 and myeloid immune cells in a representative cryosection of myocardial tissue (border zone, 3 days post-MI). CXCL8 (red) is found at the front of infiltrating leukocytes and broadly colocalizes with CD203α^+^ macrophages (green). Isolated neutrophils (yellow) can be detected by staining with the antibody (Ab) 6D10. The negative control was stained with secondary antibody only. DAPI (4′,6-diamidino-2-phenylindole) was used for nuclear counterstaining (blue)

In addition, analysis of selected differentially expressed genes revealed the upregulation of several chemokines that attract immune cells up to day 14 after MI compared to sham (Fig. 10a). Among others, the expression of the neutrophil chemoattractant gene *CXCL8* (Interleukin-8) was significantly upregulated in the porcine infarct core on days 3, 7, and 14. We further validated the presence of CXCL8 protein in our myocardial samples by immunofluorescence microscopy and found that CXCL8 expression was mainly confined to the border zone (Fig. 10b). CXCL8 colocalized with macrophages (CD203α^+^ cells in our staining), but other cells also showed expression. Neutrophils stained with the 6D10 antibody in pigs could also be detected in this region.

### Flow cytometric immunophenotyping of leukocytes from cardiac tissue

**Fig. 11:**
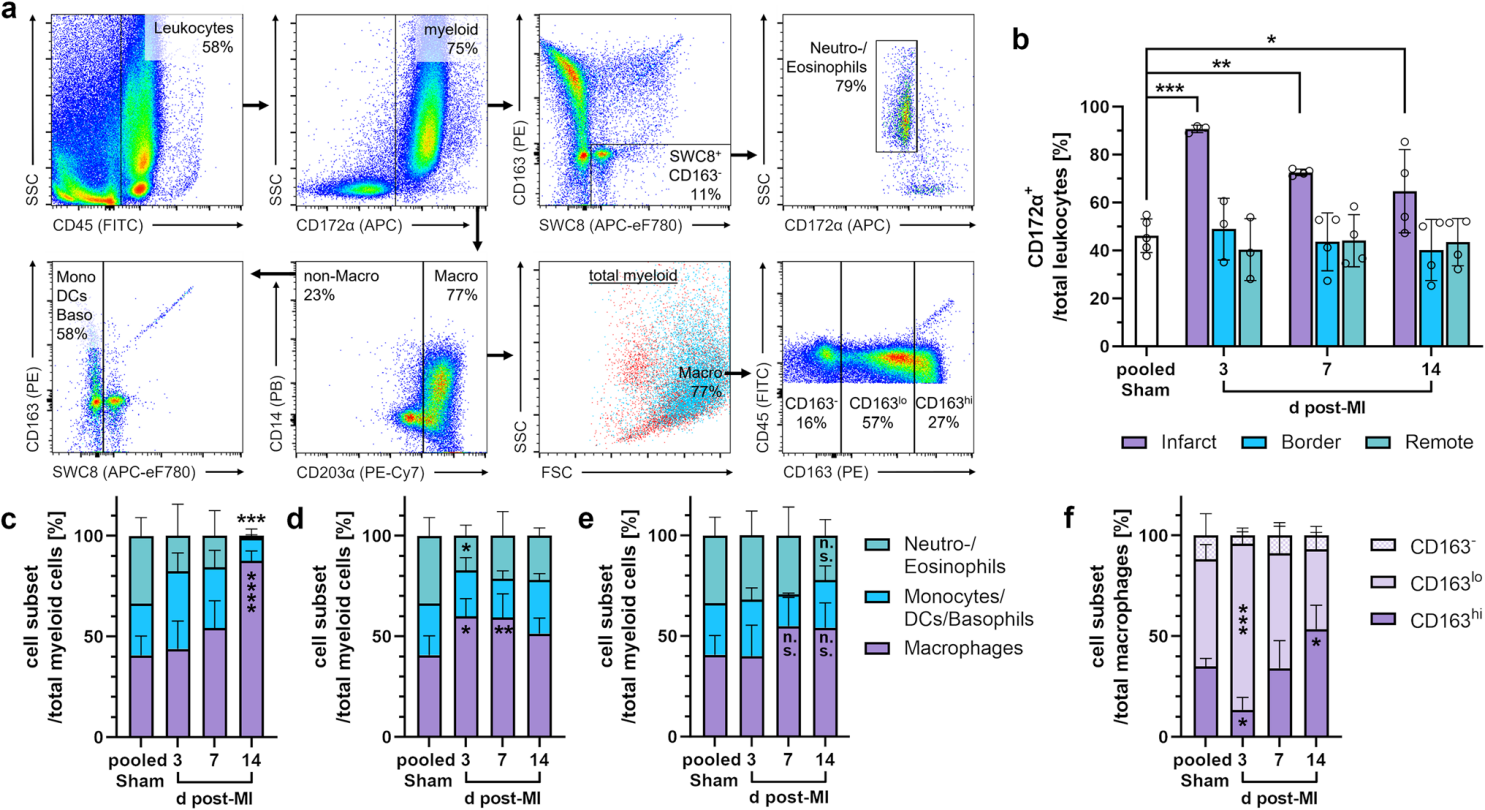
Flow cytometry of myeloid immune cells from the pig heart. **a** Sequential gating strategy for immunophenotyping of myeloid cells. All events were pre-gated on viable (Zombie Aqua^−^) singlets. **b** Proportion of myeloid (CD172α^+^) cells among total leukocytes from the different regions at the indicated points in time (d = days) post-MI or from sham hearts. **c**–**e** Proportions of myeloid cell subsets from c the infarct core, d the border zone, or e the remote zone (*n* = 3–4 each, except *n* = 2 for Monocytes/Dendritic cells (DCs)/Basophils in pooled sham). **f** CD163 surface expression on macrophages from the infarct core (*n* = 3–4). Mean ± SD; *p < 0.05, **p < 0.01, ***p < 0.001, **** p < 0.0001 vs. pooled sham (two-way ANOVA with Dunnett’s multiple comparisons test)

Among the CD45^+^ leukocytes isolated via MACS from the infarct core, flow cytometry (Fig. 11a) revealed myeloid (CD172α^+^) cell predominance across all points in time compared to leukocytes from the other sampling regions or from sham hearts (Fig. 11b). This was particularly pronounced in the early post-infarction phase (mean 91% myeloid cells among total leukocytes on day 3 post-MI).

We found that the proportion of mature macrophages (CD203α^+^) among myeloid cells in the infarct core doubled from day 3 to day 14 after MI (Fig. 11c), whereas granulocyte (SWC8^+^ CD163^−^ SSC^hi^) frequency substantially decreased, ultimately dropping to only 1% at 14 days post-MI. Outside the infarct core, an early peak in macrophages within the border zone was noticeable (Fig. 11d). In the remote zone, however, we observed no significant changes in myeloid cell composition over time (Fig. 11e). Within the mixed population of CD203α^−^ SWC8^−^ myeloid cells, comprising monocytes, dendritic cells (DCs), and basophils, our antibody panel could not achieve further differentiation. In our flow cytometric analyses, most of the CD203α^+^ macrophages isolated from the myocardium costained for CD163 (Fig. 11a). Nonetheless, compared to the remote zone or sham hearts, the infarct core showed a notably clear predominance of CD163^lo^ macrophages at the earliest point in time, whereas 14 days after MI, the CD163^hi^ subpopulation prevailed (Fig. 11f).

**Fig. 12:**
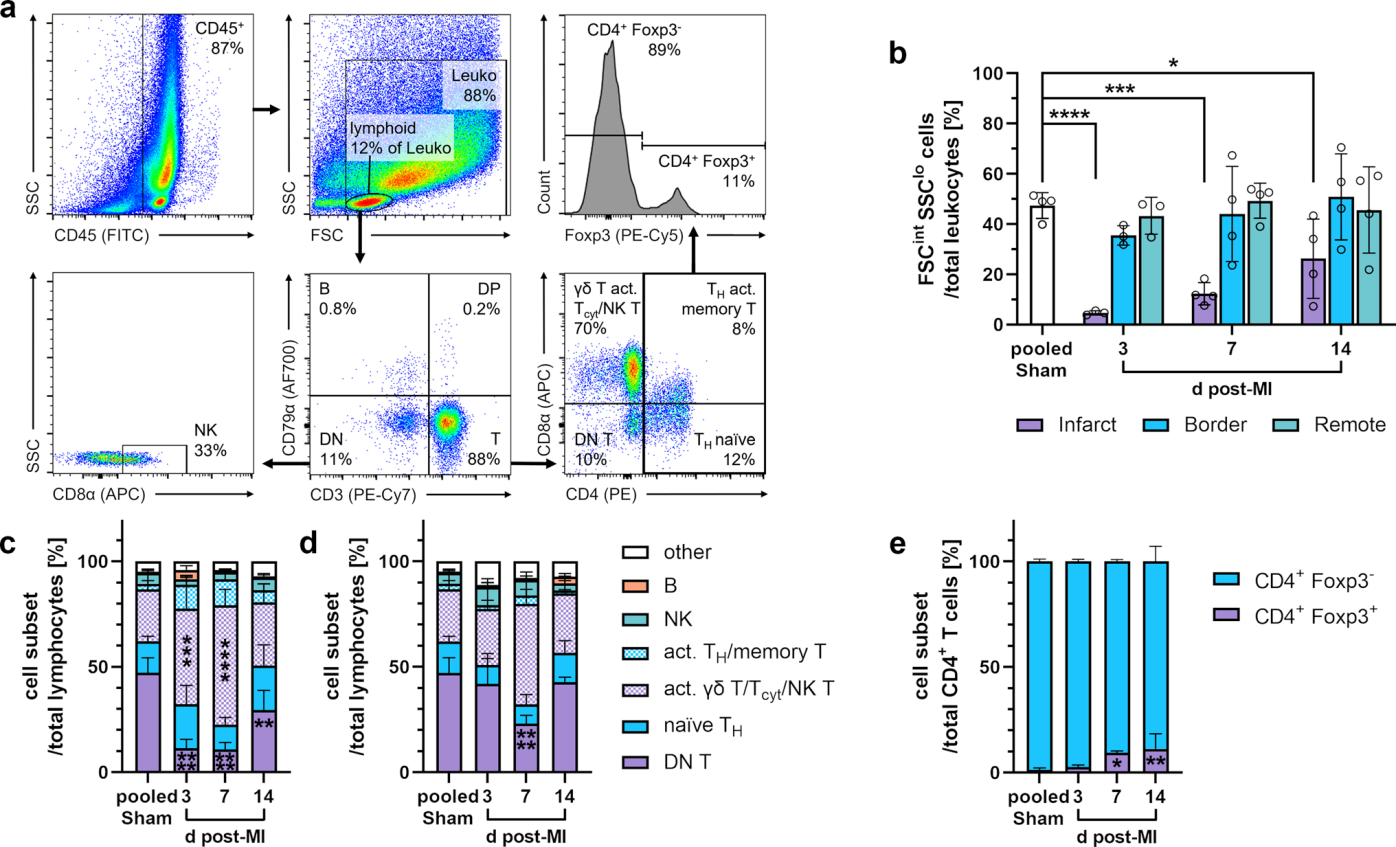
Flow cytometry of lymphocytes from the pig heart. **a** Sequential gating strategy for immunophenotyping of lymphocytes. Cells were fixed and permeabilized. All events were pre-gated on viable (Zombie Aqua^−^) singlets. DN (T): double-negative (T) cells, DP: double-positive cells. **b** Proportion of lymphocytes among total leukocytes from the different regions at the indicated points in time (d = days) post-MI or from sham hearts. **c**–**d** Proportions of lymphocyte subsets from c the infarct core or d the remote zone (*n* = 3–4 for each). **e** Foxp3 intracellular expression on CD4^+^ T cells from the infarct core (*n* = 3–4). Mean ± SD; *p < 0.05, **p < 0.01, ***p < 0.001, ****p < 0.0001 vs. pooled sham (two-way ANOVA with Dunnett’s multiple comparisons test)

In hearts from sham animals and outside the infarct core, 40–50% of all leukocytes could be classified as lymphocytes (SSC^lo^ FSC^int^) (Fig. 12a, b), most likely due to some remaining intravascular leukocytes. In contrast, the lymphocyte fraction in the infarct core appeared substantially reduced, although increasing, across all points in time. Within this fraction, T cells (CD3^+^) were the predominant subset (Fig. 12c, d).

In the myocardium, we observed a relative expansion of the combined population of activated γδ T, T_cyt_, and NK T cells (CD3^+^ CD4^−^ CD8α^+^), while *bona fide* naïve γδ T cells (CD3^+^ CD4^−^ CD8α^−^) were correspondingly reduced in the infarct core on day 3 and particularly on day 7 post-MI, compared to sham samples (Fig. 12c). At the latter point in time, this pattern also occurred in the other myocardial regions, even the remote zone, vs. sham control (Fig. 12d). In addition, Foxp3^+^ T_reg_ among CD3^+^ CD4^+^ cells significantly increased in the infarct core on days 7 and 14 after MI vs. sham (Fig. 12e).

### Flow cytometric analysis of extramedullary myelopoiesis in the spleen

**Fig. 13:**
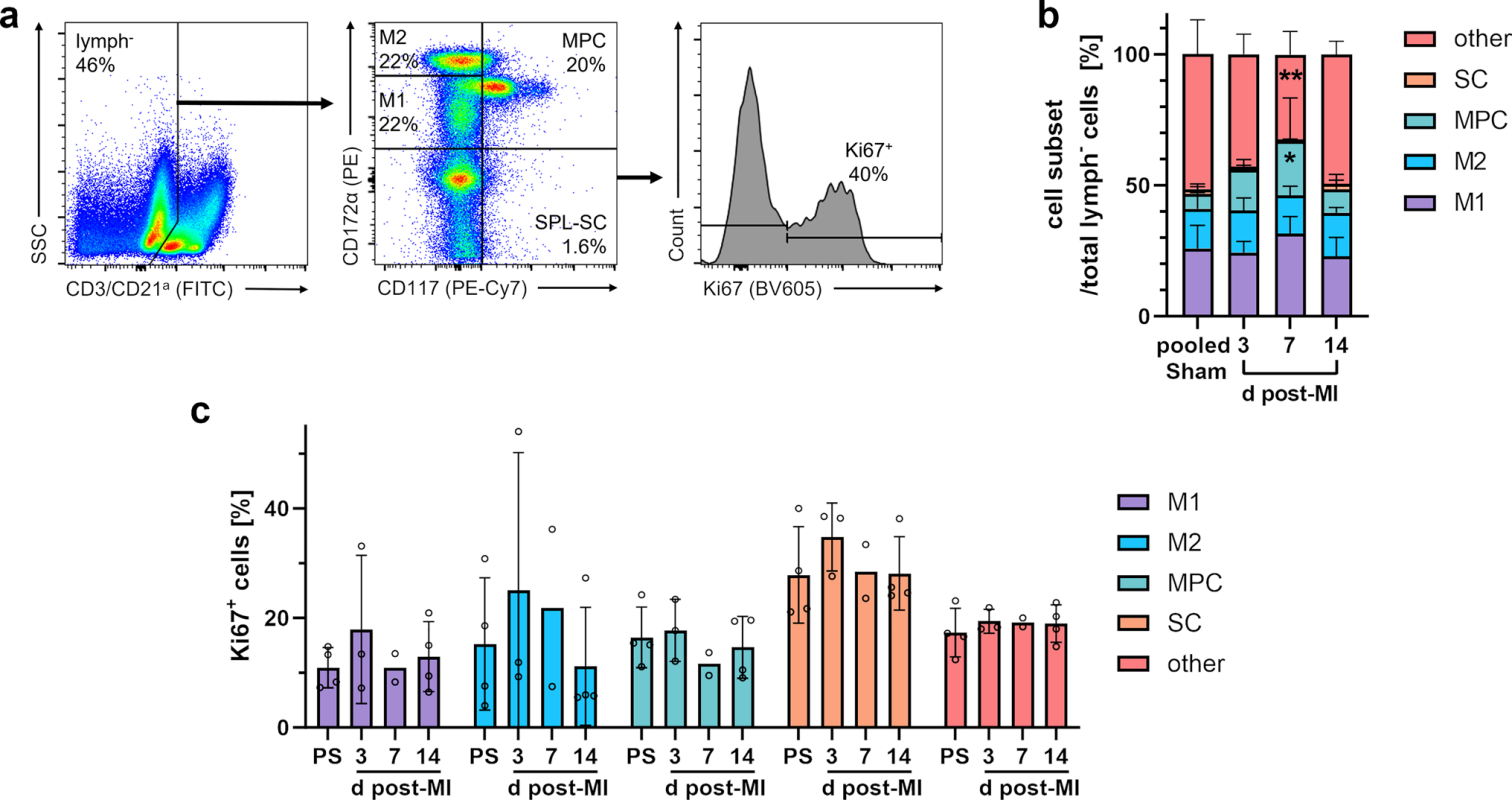
Flow cytometry of stem and myeloid progenitor cells from the porcine spleen. **a** Sequential gating strategy for the identification of stem cells (SC), myeloid progenitor cells (MPC) and myeloid cell subsets (M1, M2) among lymphoid lineage markers CD3 and CD21^a^ negative (lymph^−^) spleen (SPL) cells. Cells were fixed and permeabilized. All events were pre-gated on viable (Zombie Aqua^−^) singlets. **b** Proportions of spleen cell subsets among lymphoid lineage marker negative cells at the indicated points in time (d = days) post-MI and from sham animals (*n* = 3–4 each). **c** Proliferation rate of the individual spleen cell subsets as determined by intracellular Ki67 expression (PS: pooled sham). Mean ± SD; *p < 0.05, **p < 0.01 vs. pooled sham (two-way ANOVA with Dunnett’s multiple comparisons test)

Next, we focused our flow cytometric analysis on the porcine spleen as a potential site of MI-induced extramedullary myelopoiesis contributing to myeloid cell supply during cardiac healing [39]. Among spleen cells, we could identify stem cells (SC: CD117^+^ CD172α^−^) as well as myeloid progenitor cells (MPC: CD117^+^ CD172α^lo−int^). Furthermore, we were able to distinguish two additional myeloid cell subsets, M1 (CD117^−^ CD172α^lo−int^) and M2 (CD117^−^ CD172α^hi^) (Fig. 13a), which likely represent earlier and later stages of mono-/granulocytic cell maturation. In our analyses, we found a significant expansion of MPC in spleens from infarcted pigs compared to organs from sham animals on day 7 (Fig. 13b). Overall, only a very small SC fraction could be detected.

### Flow cytometric immunophenotyping of lymphocytes from heart-draining lymph nodes

**Fig. 14:**
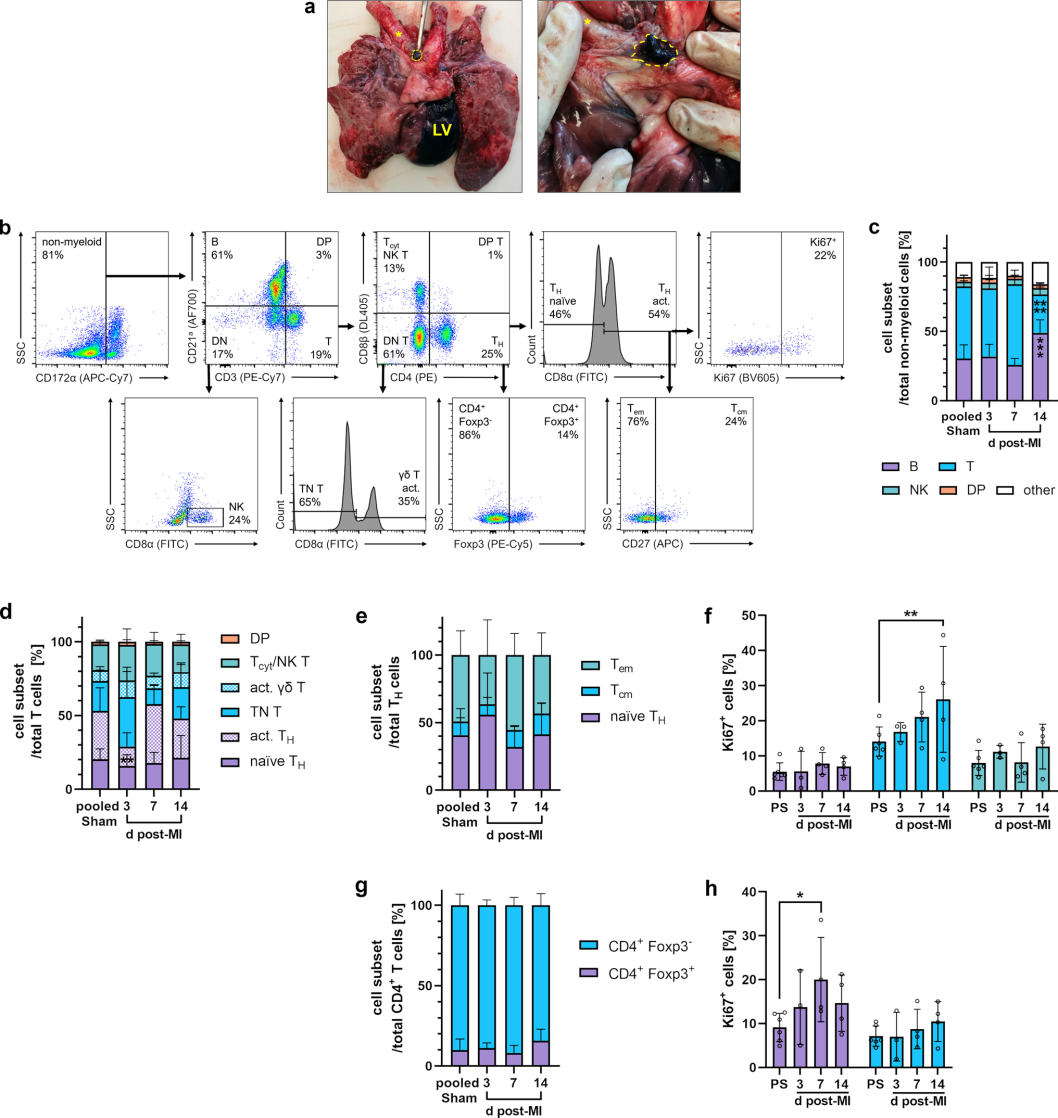
Flow cytometry of lymphocytes in heart-draining lymph nodes of the pig. **a** Autopsy preparation of the porcine cardiopulmonary system. Mediastinal heart-draining lymph nodes (yellow dashed line) can be identified in close proximity to the trachea (yellow asterisk) after toluidine blue injection into the left-ventricular (LV) myocardium. **b** Sequential gating strategy for immunophenotyping of lymphocytes. Cells were fixed and permeabilized. All events were pre-gated on viable (Zombie Aqua^−^) singlets. DN (T): double-negative (T) cells, DP (T): double-positive cells, TN T: triple-negative T cells. **c** Proportions of lymphocyte subsets at the indicated points in time (d = days) post-MI (*n* = 3–4) and from sham animals (*n* = 6). **d** Proportions of T-cell subsets (*n* = 3–4 for post-MI, *n* = 6 for pooled sham). **e** Proportions of T_H_ subsets (*n* = 3–4 for post-MI, *n* = 6 for pooled sham). f Proliferation rate of the individual T_H_ subsets as determined by intracellular Ki67 expression (PS: pooled sham). **g** Foxp3 intracellular expression on CD4^+^ T cells (*n* = 3–4 for post-MI, *n* = 6 for pooled sham). **h** Proliferation rate of the individual CD4^+^ Foxp3^±^ cell subsets. Mean ± SD; *p < 0.05, **p < 0.01, ***p < 0.001, ****p < 0.0001 vs. pooled sham (two-way ANOVA with Dunnett’s multiple comparisons test)

After dye injection into the infarct region of a pig heart in situ, we could confirm the typical tracheobronchial location of the primary heart-draining lymph nodes (Fig. 14a), where the adaptive immune response to antigens from the injured myocardium takes place [28]. We found that they were often enlarged after MI, consistent with some interindividual heterogeneity in the adaptive immune response to cardiac injury. Flow cytometric analysis of the major lymphocyte subsets in the lymph nodes (Fig. 14b) revealed a significantly higher frequency of B cells and a concomitant reduction in T cells on day 14 post-MI compared to sham (Fig. 14c). Within CD4^+^ CD8β^−^ T_H_, we found the activated/memory (CD8α^+^) cell subset decreased on day 3 after MI (Fig. 14d). There were no substantial changes between the relative compositions of effector/central memory T cells (T_em_, T_cm_) and naïve T_H_ cell subsets (Fig. 14e). In addition, T_cm_ (CD4^+^ CD8β^−^ CD8α^+^ CD27^+^) proliferated significantly more than sham on day 14 (Fig. 14f). Also, within CD4^+^ CD8β^−^ cells, *bona fide* T_reg_ could be identified by Foxp3 expression (Fig. 14b, g). Ki67 expression indicated that these CD4^+^ Foxp3^+^ T cells had increased proliferation, compared to sham, 7 days after MI (Fig. 14h).

## Discussion

**Fig. 15:**
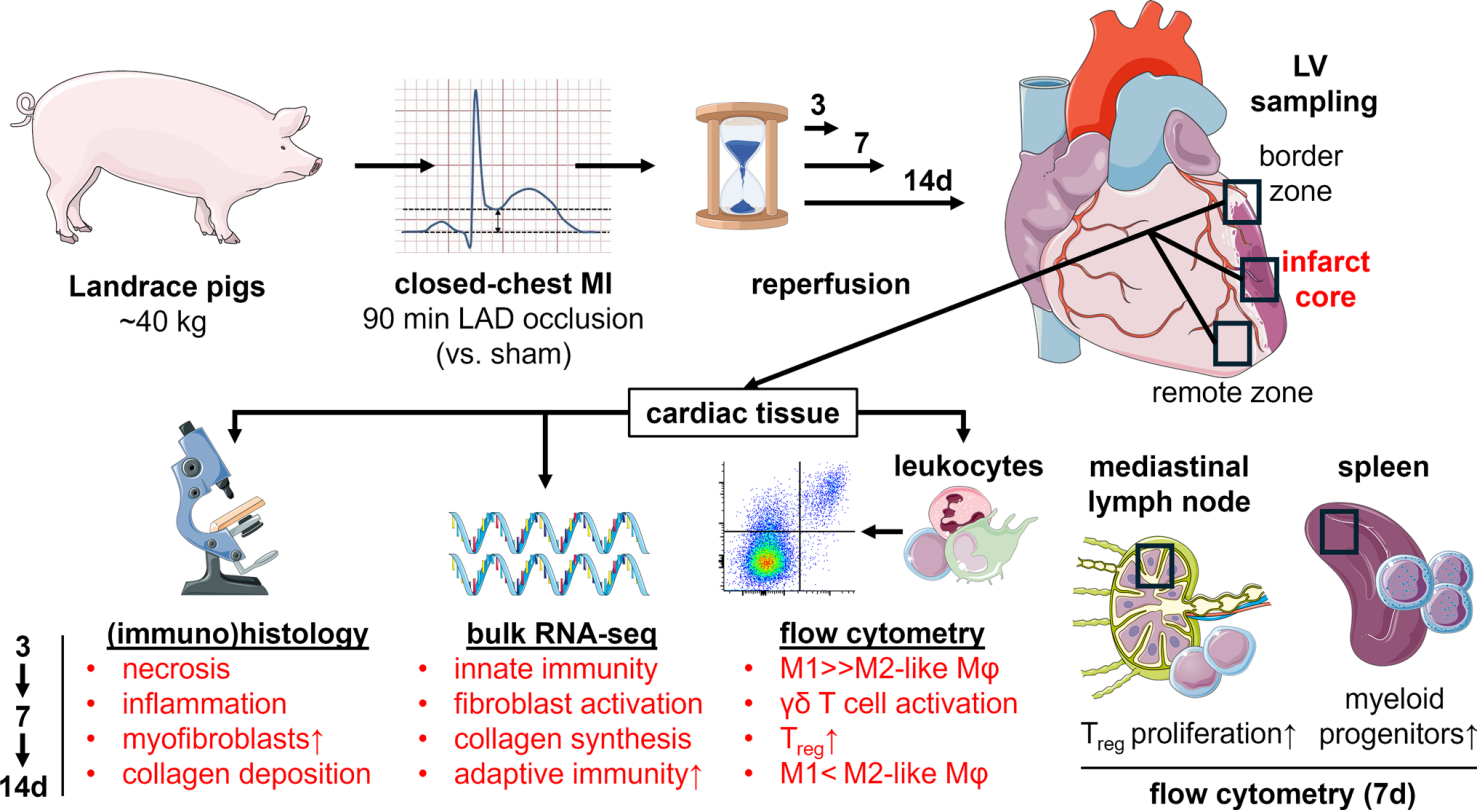
Overview: experimental setup and key results. Landrace pigs weighing an average of 40 kg were subjected to either closed-chest myocardial infarction (MI) through interventional balloon occlusion of the left anterior descending artery (LAD) for 90 min or a corresponding sham procedure. After a reperfusion period of 3, 7, or 14 days (d), the animals were sacrificed and cardiac tissue samples were obtained from different left-ventricular (LV) wall regions of the explanted hearts. The samples were further processed for (immuno)histology, bulk-tissue RNA sequencing (RNA-seq), and flow cytometry (of isolated CD45^+^ leukocytes) to investigate the spatiotemporal dynamics of immune-mediated inflammatory and healing processes in the infarcted pig heart. Key findings related to the infarct core over time are highlighted in red. For extended immunological characterization, tissue from heart-draining mediastinal lymph nodes and spleen was also analyzed by flow cytometry. Mφ: macrophages, T_reg_: regulatory T cells; ↑ increased, >  > considerably greater than. The figure was partly generated using Servier Medical Art, provided by Servier (CC BY 3.0) as well as the image “ST_elevation_illustration.jpg” by PeaBrainC, via Wikimedia Commons (CC BY-SA 4.0)

In the present study, we characterized the immune response to experimental MI induced by coronary ischemia with reperfusion in the pig. We provide, for the first time, a detailed description of the cellular protagonists driving the immune response and their spatiotemporal dynamics within 14 days after MI (Fig. 15).

Using histology and immunofluorescence microscopy, we detected mainly myeloid immune cell infiltrates that had shifted from the border zone, where they predominated on day 3, toward the infarct core on day 7 post-MI. Although necrotic tissue replacement, including increased collagen deposition, had markedly progressed on day 14, residual necrotic areas surrounded by leukocytes could still be seen. This was reflected by our transcriptome data which, at that point in time, showed ongoing inflammatory activity in parallel to a strong pro-fibrotic signature.

### Interspecies comparison of healing dynamics after MI

Qualitatively and temporally similar processes have been described in infarcted human myocardium with respect to immune cell infiltration and scar formation [8, 51]. In contrast, reperfused infarcts in mouse hearts already showed nearly complete replacement of necrotic cardiomyocytes with granulation tissue and immune cells after only 3 days. On day 7 and thereafter, thinning and progressive cell loss were evident in the murine infarct zone [10, 13]. In human infarcts, gradual replacement of granulation tissue with more compact collagen begins after 4–6 weeks and a dense, hypocellular scar is fully formed 2–3 months post-MI [8, 51]. While in mice the immune response in reperfused vs. permanent MI is accelerated and early inflammation is enhanced by reperfusion, the healing process is also faster [84]. We can assume that this likewise applies to pigs. Therefore, permanent occlusion in these animals might yield different results especially regarding leukocyte kinetics in the injured heart.

After MI, replacement of necrotic myocardium by a fibrous scar is essential to maintain structural integrity of the ventricular wall. Characteristically and exclusively during the proliferative phase of myocardial healing, α-SMA^+^ myofibroblasts derived from activated fibroblasts migrate into the infarct core where they synthesize extracellular matrix components and mediate repair [22, 31]. A key regulator of these events is the local tissue environment, which is profoundly shaped by leukocytes [44]. The reduction of vimentin^+^ α-SMA^−^ fibroblasts and endothelial cells that we observed in the infarct core at day 3 compared to the other myocardial regions can be attributed to necrosis. We found these cells predominantly in the border zone, whereas vimentin^+^ α-SMA^+^ myofibroblasts increasingly infiltrated the infarct core. This basically mirrors the situation in humans, where myofibroblasts appear in the border zone granulation tissue on day 4 after MI and then were highly abundant in the infarct region from day 14 on [79]. In mice, by contrast, myofibroblasts in the infarct peaked as early as 3 days after reperfusion, with a sharp decline in cell numbers after day 7 [10, 13].

Table 1 provides an overview of relevant own findings in the pig MI model compared to published results on immune cell kinetics and healing in the infarcted heart as well as the involvement of extracardiac lymphatic tissues in humans and mice. It is important to note that some of the reported differences between species may also be due to methodological factors.

**Table 1:** Cross-species comparison of the dynamics and characteristics of cardiac healing and extracardiac lymphatic tissue involvement after myocardial infarction (MI)

	Pig (Reperfused MI)	Human	Mouse
**~ 3d post-MI**			
Phase of cardiac repair	Inflammatory phase	Inflammatory phase [37]	(Early) Proliferative phase [10, 13]*
Heart: Microscopic aspect	IC: CM necrosis, BZ: Mainly myeloid immune cells	IC: CM necrosis [8, 51], BZ: CD14^+^ cells [37]	CM necrosis almost entirely replaced with granulation tissue/leukocytes [10, 13]*
Heart: Myofibroblasts	BZ: Starting accumulation	BZ: Starting accumulation (day 4) [79]	Peaking in the infarct [10, 13]*
Heart: Granulocytes	IC, BZ: relatively few	Peaking (days 1–3) [8, 51]	↓ (Peak on day 1) [13, 84]*
Heart: Macrophages	IC: Starting accumulation, M1-like >> M2-like, BZ: Peaking	Starting accumulation [8, 51]	Peaking [10, 13, 84]*, M1 (peaking) > M2 [84]*
Heart: Lymphocytes	IC: Few, evidence of γδ T cell activation	Starting accumulation [8, 51]	Peaking [84]*, Including T_reg_ [81]*
Mediastinal lymph nodes	Activated/memory T_H_ ↓	N/A	Activated CD4^+^ T cells ↑, T_reg_ ↑ [28]
**~ 7d post-MI**			
Phase of cardiac repair	Proliferative phase	Proliferative phase [37]	(Late) Proliferative phase [10, 13]*
Heart: Microscopic aspect	IC: Granulation tissue, mainly myeloid immune cells, CM necrosis, BZ: Granulation tissue	IC: Granulation tissue, CM necrosis [8, 51], numerous CD14^+^ cells [37], BZ: Granulation tissue [8, 51]	Thinning, progressive cell loss [10, 13]*
Heart: Myofibroblasts	IC: Starting accumulation, BZ: Peaking	BZ: Further accumulation [79]	↓ [10, 13]*
Heart: Granulocytes	IC: ↓ BZ: Few	↓↓ [8, 51]	↓↓ [10, 13, 84]*
Heart: Macrophages/ monocytes	IC: Ongoing accumulation, M1-like > M2-like BZ: (still) peaking	Peaking (days 5–10) [8, 51], Inflammatory > other monocytes [37]	↓ [10, 13, 84]*, M2 >> M1 [ 84 ]*
Heart: Lymphocytes	IC: T_reg_↑, All regions: evidence of γδ T cell activation	Peaking (days 5–10) [8, 51], including T_reg_ (days 5–14) [59]	↓, Treg still relatively elevated [81, 84]*
Mediastinal lymph nodes	T_reg_ proliferation ↑	Size ↑, T cells (CXCR4^+^) ↑ [59]	Cellularity ↑, CD4^+^ T cell proliferation ↑ [28], T_reg_ peaking, activated T_reg_ ↑ [78]
Spleen	MPC ↑	N/A	MPC peaking (day 6) [39]
**~ 14d post-MI**			
Phase of cardiac repair	Proliferative phase	Proliferative phase [37]	Maturation phase [10, 13]*
Heart: Microscopic aspect	IC: Fibrovascular granulation tissue, isolated (mainly myeloid) immune cells, clusters around residual necrotic CM, BZ: fibrovascular granulation tissue	Fibrovascular granulation tissue, (early) collagen, residual CM necrosis [8, 51]	Further cell loss, thinned areas, large vessels, incipient scar maturation [10, 13]*
Heart: Myofibroblasts	IC: ↑, BZ: ↓	Abundant in the infarct [79]	↓↓ [13]*
Heart: Granulocytes	IC: ↓↓↓	None [8, 51]	None (day 16) [52]
Heart: Macrophages	IC: ↑↑, M2-like > M1-like	Can persist for weeks [8, 51]	↓ (Returned to baseline) (day 16) [52]
Heart: Lymphocytes	IC: T_reg_ ↑↑	Can persist for weeks [8, 51], T_reg_ peaking (until day 14) [59]	↓↓ CD4^+^ T cells, Including T_reg_ [35]
Mediastinal lymph nodes	B cells ↑, T cells ↓, T_cm_ proliferation ↑	N/A	N/A

Table of relevant own findings in the porcine MI model compared with published results in humans and mice. *indicating data from models of reperfused MI

*BZ* border zone, *CM* cardiomyocyte(s), *CXCR4* CXC motif chemokine receptor 4, *d*  days, *IC* infarct core, *MPC* myeloid progenitor cells, *N/A* not available, *T*_cm_ central memory T cells, T_H_: T helper cells, T_reg_: regulatory T cells. >  > considerably greater than; ↑ increased, ↑↑ further increased; ↓ decreased, ↓↓ further decreased, ↓↓↓ markedly decreased

### Cardiac healing after MI as an immune-modulated process

Our transcriptome data generated from bulk RNA-seq of infarcted porcine myocardium provide insights into the time-dependent interplay between immunity and scar formation. GSEA revealed that transcripts related to cytokines that promote myofibroblast formation (e.g., *IL6*) as well as α-SMA (*ACTA2*), which is a myofibroblast marker gene, are already prominently overexpressed in the infarct core vs. sham at day 3 post-MI. In contrast, transcripts indicative of fibroblast activation (e.g., *POSTN*) and increased collagen synthesis appear later. Protein–protein interaction network plots based on bulk RNA-seq-derived gene sets mostly revealed connections between innate immunity (represented by e.g., TLR2, IL6, CCL2, and CD14) and extracellular matrix biology at days 3 and 7, whereas adaptive immunity seems to come more into play at day 14. This is exemplified by Osteonectin (*SPARC*) and TGF-β, proteins reported to mediate pro-fibrotic effects of T_reg_ in mouse myocardium [59, 81]. In addition, a recently published meta-analysis including STRING-based analysis of protein–protein interactions showed that myocardial fibrosis-related pathways in failing hearts are promoted by T-cell-mediated immune responses. Notably, fibromodulin (*FMOD*), which is also represented in our day 7 and 14 interaction network, was identified as a hub gene across different databases [72].

In principle, our findings are well compatible with results from a similar porcine MI model [74], where tissue gene expression analysis by qPCR revealed early activation of the pro-inflammatory TLR-4 pathway of the innate immune response, involving upregulation of key downstream mediators, such as *TNF*, *IL6*, and ultimately *NFKB1* within hours of reperfusion. Pro-fibrotic *TGFB1* gene expression peaked on day 1 post-MI, while (immuno)histochemistry showed a subsequent gradual increase in fibroblasts and collagen from day 3 on.

Given the central role of immunity as a regulator of cardiac healing, it was necessary to study the leukocyte composition in the injured myocardium.

### Myeloid cell infiltration and differentiation in the infarcted heart

Flow cytometric immunophenotyping enabled us to gain more detailed insights into the composition of immune cell subsets in our porcine MI model. Among myeloid cells, we likely missed the initial peak of neutrophils. Macrophages appeared abundant in the border zone already on day 3 and their proportion significantly increased in the infarct core until day 14. Accordingly, Vilahur et al. [74] observed the accumulation of (unspecified) leukocytes in H.E.-stained infarcted porcine myocardium as early as 90 min after reperfusion, peaking on day 3, whereas immunohistochemistry showed a substantial increase in macrophages on day 3, with a further rise on day 6. Overall, this correlates well with the situation in human infarcts, where granulocytes are mainly present on days 1–3, decreasing thereafter, and macrophage accumulation starts on day 3, reaching a (sustained) maximum on days 5–10 post-MI [8, 51]. In reperfused murine infarcts, however, neutrophils were most prevalent on day 1 and macrophages on day 3, respectively, with a considerably lower frequency from day 7 onwards [13, 84].

Macrophages play a central role in mediating inflammation and promoting healing after MI; their specific roles depend on their functional phenotype [54]. There is a serious lack of well-defined surface markers for phenotypically characterizing macrophages in pigs, and various in vitro polarization studies on this topic have reported partly divergent results [9, 62, 65]. Our flow cytometric analyses demonstrated that CD163^lo^ macrophages markedly predominated in the infarct core on day 3 post-MI vs. sham, whereas the CD163^hi^ subpopulation prevailed at 14 days. CD163 upregulation following in vitro exposure to either anti-inflammatory IL-10 or dexamethasone was reported for pig macrophages [7, 65]. Hence, the observed shift between macrophage subpopulations defined by their CD163 surface expression levels may reflect the transition from a pro-inflammatory (M1-like) to an anti-inflammatory (M2-like) phenotype during healing, as previously described in murine infarcts [84]. Furthermore, in a pig model that is methodologically comparable to ours, Bönner et al. [4] imaged the accumulation of monocytes/macrophages in the infarct region using cardiac ^19^F MRI on day 6 after reperfused MI. Complementary immunofluorescence microscopy revealed almost equal proportions of CD163^lo^ (M1-like) and CD163^hi^ (M2-like) macrophages in the infiltrates, supporting the notion that the turning point between inflammation and repair in the porcine heart is reached approximately 1 week post-MI.

### MI-induced splenic myelopoiesis

Most macrophages found in the injured heart are derived from infiltrated monocytes, for which the spleen serves as an important reservoir. Its replenishment in this context is achieved through extramedullary (emergency) myelopoiesis by progenitor cells released from the bone marrow that have seeded the spleen [16]. The organ’s role in the immune response after MI is ambivalent. On the one hand, the spleen fuels the initial inflammatory stage [82] by providing large numbers of monocytes via the bloodstream to the infarcted myocardium, which then differentiate into pro-inflammatory macrophages. In this way, splenic monocyte supply might contribute to adverse remodeling and the development of heart failure. On the other hand, pro-inflammatory macrophages ensure sufficient phagocytosis of debris and prepare the ground for the subsequent healing process, which is again mediated by monocyte-derived, albeit anti-inflammatory macrophages. Monocyte depletion in this phase after MI might therefore even impair cardiac healing by preventing a timely resolution of inflammation and eventually lead to adverse remodeling [24]. Moreover, a study in pigs with acute myocardial ischemia–reperfusion injury highlights a central role of the spleen in transducing the cardioprotective effects of preceding brief ischemic periods in distant body regions (“remote ischemic preconditioning”) via the release of an unidentified humoral factor, involving vagal activation of the organ [43]. Since the hematopoietic stem and progenitor potential of porcine CD117 (c-kit)^+^ cells has been demonstrated both in vitro and in vivo [21], we combined CD117 and CD172α to identify stem cells and myeloid progenitor subsets. Using this strategy, we found first evidence of increased myelopoiesis in response to MI in the spleen of pigs. Hence, this approach will allow to study the impact of immunotherapeutic interventions on splenic myelopoiesis.

### Post-MI lymphocyte dynamics

In one of the few descriptive studies in pigs that involved post-MI flow cytometric immunophenotyping [45], peripheral blood lymphocytes from 1 h after ischemia–reperfusion were analyzed. Major findings were an increased CD4^+^/CD8^+^ ratio and a reduction of effector/memory (CD27^−^) in favor of naïve (CD27^+^) CD8^+^ T cells compared to pre-MI levels, indicating an early impact on the adaptive immune system.

Our analyses, focusing on the heart at later points in time after MI, showed that the relatively small lymphocyte fraction from porcine infarcts, which consisted mainly of T cells, expanded over time. Accordingly, in the infarcted human myocardium, lymphocytes appear on day 3, reach a maximum between 5 and 10 days post-MI, and can persist for several weeks [8, 51]. In murine infarcts, by contrast, the frequencies of all major lymphocyte populations were highest after 3 days of reperfusion and then returned to sham levels [84]. Our flow cytometric subset analysis revealed a relative increase of CD4^−^ CD8α^+^ T cells in the porcine infarct core, particularly on days 3 and 7 after MI. Although we were not able to distinguish between T_cyt_, activated γδ T, and NK T cells within this population, the concurrent reduction of the *bona fide* naïve γδ T-cell subset suggests that activated γδ T cells particularly become more prevalent after MI. High γδ T-cell frequency is a peculiarity of the porcine immune system, especially in younger animals. A large knowledge gap remains regarding the functions of these cells, though in vitro stimulation assays have revealed their capacity for pro-inflammatory cytokine (IFN-γ, TNF, and IL-17A) production [60]. Notably, in murine MI, heart-infiltrating γδ T cells but not CD4^+^ T_H_ were identified as major producers of IL-17A, responsible for functional deterioration by sustained inflammation, cardiomyocyte apoptosis, and fibrosis during remodeling [40, 85].

Additionally, we detected a higher proportion of Foxp3^+^ *bona fide* T_reg_ among CD4^+^ T cells in the infarct core at 7 and 14 days post-MI. This corresponds to earlier findings in human autopsy samples, where CD4^+^ Foxp3^+^ cells peaked in the infarct during the proliferative phase (5–14 days) after MI [59]. In reperfused murine infarcts, however, T_reg_ were already most prevalent on day 3 [81]. Moreover, our flow cytometric approaches strongly indicated that T cells were activated: our analyses showed increased proliferation of CD4^+^ T cells, including Foxp3^+^ cells, in heart-draining lymph nodes in response to MI. These observations align with the activation processes in mediastinal lymph nodes after MI in human patients [59]. Since T_reg_ stimulation is considered a promising target for future immunotherapeutic approaches after MI [77], it appears particularly relevant that T_reg_ post-MI kinetics in the pig model are comparable to those in humans.

With respect to the other lymphocyte populations in the porcine heart-draining lymph nodes, the transient decrease we found in the activated/memory T_H_ subset on day 3 after MI may reflect emigration of these cells. This could be part of an early adaptive immune response in the lymph nodes involving myocardial antigen-induced activation of autoreactive CD4^+^ T cells, which have been shown to recirculate and eventually infiltrate the infarcted myocardium in mouse models [28, 59]. Moreover, the significantly higher frequency of B cells we detected at day 14 may be indicative of clonal expansion in the context of a germinal center response as described in mice at a comparable stage of post-MI healing [23]. However, there is a need for more focused research to determine whether these processes do indeed occur in pigs as well.

### Technical and methodological limitations

Note that these applied technical approaches do have limitations: Because of the large size of a pig heart, it is not feasible to analyze the entire infarcted tissue. Hence, to account for the significant spatial heterogeneity within the infarct core and border zone, representative sampling from these regions is critical. Typically, they can be precisely localized using 2,3,5-triphenyltetrazolium chloride (TTC) staining as the gold standard to distinguish viable from infarcted myocardium, complemented by blue dye coloration to delineate the area at risk [5]. In this study, however, staining was not performed to avoid interfering with further histological and molecular biological analysis of the tissue. Therefore, the sampled regions were delineated based on visual criteria only. Moreover, our immunofluorescence microscopy shows that most leukocytes have extravascular locations. However, even thorough antegrade coronary perfusion will leave some blood leukocytes which are often marked in mice by an anti-CD45 antibody in the perfusate. Due to the required volume for pig hearts, this approach would be very costly and could not be implemented by us.

Flow cytometric phenotyping of immune cells allows the delineation of leukocyte subsets and differentiation states in tissues and has become a standard immunological technique in mice and humans. In pigs, however, both the number of well-defined leukocyte markers itself and even more, so the repertoire of available antibodies for this specific application are relatively limited [11, 60]. With the antibody portfolio described here, we were able to identify the most relevant immune cell subsets from porcine hearts by flow cytometry. Some details can still be improved, but in many cases, no further differentiation is possible at this time. For instance, we considered CD3^+^ CD4^−^ CD8α^±^ CD8β^−^ leukocytes from porcine lymph nodes as *bona fide* γδ T cells. Adding antibodies against the γδ T-cell receptor and CD2 would allow for direct identification and more refined subset characterization of these cells. Regarding porcine macrophages, however, we still lack markers to discriminate resident from monocyte-derived cells or to characterize macrophage differentiation states. Macrophages within the mouse heart are partitioned into CCR2^−^ and CCR2^+^ subsets reflecting resident and monocyte-derived origins. Likewise, in the human myocardium, one can find CD64^+^ CD68^+^ CCR2^−^ and CD64^+^ CD68^+^ CCR2^+^ macrophages [2]. For pigs, to the best of our knowledge, there is no antibody available with reactivity against CCR2. Moreover, whereas the selectivity of our flow cytometric analyses from heart tissue can be readily enhanced for T_cyt_ and NK T cells by adding an anti-CD8β antibody to the panel, properly differentiating between monocytes and DCs remains challenging, again due to the lack of mutually exclusive markers or appropriate antibodies. In general, the limited availability of antibodies to delineate all leukocyte subsets and their differentiation states can only be overcome by single-cell transcriptomic approaches, which have already proven very useful for dissecting cardiac heterocellularity in general [46]. Further, we found that accurate quantification of absolute leukocyte counts was impracticable, because tissue processing inevitably resulted in a loss of cells that could not be readily estimated. We also noticed differences in the efficacy of tissue digestion and subsequent MACS enrichment, as well as in the viability of recovered leukocytes between various animals, points in time, and myocardial regions.

In addition, there are several limitations associated with the porcine MI model per se, such as the susceptibility of the animals to malignant arrhythmias associated with infarct induction and their relatively expensive acquisition and maintenance. Furthermore, a major characteristic of common farm pigs is their enormous growth that makes studies in older animals or long periods of follow-up challenging. Therefore, for reasons of practicability, only young female Landrace pigs were used in this study. These animals are still in the growth phase and can be roughly compared to 6–7-year-old human beings [71]. However, their cardiovascular parameters (e.g., heart rate, blood pressure) and anatomy (e.g., organ sizes) already correspond well to those of adult humans, or even match them ideally as in the case of the heart-to-body-weight ratio, where older pigs appear less suitable [38]. On the other hand, since the animals have not yet reached sexual maturity, at least gender-specific hormonal influences can be largely excluded. Notably, a more recent prospective study even on sexually mature Göttingen minipigs found no evidence for a presumed protective effect of female sex in experimental ischemia–reperfusion injury [34].

It must be kept in mind that the pathophysiology of experimental MI and the subsequent immune response in a young, healthy, female pig does not directly reflect the situation in typically much older, human patients of both sexes with pre-existing risk factors and comorbidities, often under medical treatment. Therefore, refinement of the animal model to further approximate human clinical reality, particularly with respect to the effects of aging, cardiovascular risk factors, and concomitant diseases, would be desirable for future immunotherapy studies. Various strains of pigs (e.g., minipigs with much more limited weight gain with age) as well as genetically modified animals have already been described and appear useful for this purpose [1, 26, 48].

### The value of the pig MI model for translational immunotherapy studies

As advances in cardioimmunology continue to shape our understanding of the immune system’s role in heart disease, we also urgently need to move forward on the translational pathway. Numerous studies, mostly in mice, have described novel potential targets for immunotherapy in this context. Yet, very few such approaches have been subsequently evaluated in clinically relevant large animal models, not least due to the lack of knowledge and tools to study pig immunology in cardiovascular health and disease.

Even considering the limitations mentioned above, the pig species in general has important strengths that make it an excellent large animal model in this area of research. Pigs are relatively easy to breed and maintain. Their cardiovascular system exhibits remarkable anatomical and (patho)physiological similarities to humans. It is amenable to a wide variety of interventions and measurements, many of which have parallels in human clinical application. Because of the size of the porcine heart, it is more feasible than in rodents to create infarcts confined to specific regions of the organ by occluding coronary arteries and their branches. The amount of tissue that can be obtained generally allows multiple parallel analyses in different regions of a single heart [25].

Utilizing appropriate animal species is vital for the preclinical evaluation of novel immunotherapeutic strategies. The suitability of the pig for modeling human adaptive immunity is particularly evident when considering studies in which T cells were targeted by a subclass of anti-CD28 monoclonal antibodies designated as superagonistic antibodies [3]. In a first-in-human trial, intravenous bolus infusion of the respective anti-CD28 superagonist TGN1412 (TAB08) induced an unexpected severe cytokine release syndrome (CRS) [69]. Both preclinical testing conducted on human T cells in vitro as well as in vivo applications in laboratory animals, including primates, had failed to predict this side effect. Later, having generated a corresponding superagonist with reactivity for porcine CD28, we found that its infusion into healthy Landrace pigs also caused CRS [73]. In fact, these animals are the only species described so far in which the same dose of CD28 superagonist as in humans was sufficient to induce CRS, thereby underscoring the suitability of the porcine model for such research. Moreover, *CXCL8* expression detected in the infarcted porcine myocardium exemplifies the value of this species for translational cardioimmunological studies. Macrophages expressing the neutrophil chemoattractant CXCL8 have been described in both terminally failing and infarcted human hearts, but this cytokine is not expressed in mice [36, 58]. The pig model would therefore provide a unique opportunity to study the functional significance of CXCL8 in the heart.

To summarize, we describe for the first time how the cellular immune response evolves in parallel with healing processes after experimental MI in pigs and provide exemplary data as well as methodological advice. Our findings reveal a marked similarity to the human situation in various key aspects, highlighting the utility of the pig MI model for cardioimmunological studies. We hope that our work can advance the field toward clinical translation by serving as a helpful initial reference point and by encouraging other researchers to adopt and refine these tools for their future projects.

### Supplementary Information

Below is the link to the electronic supplementary material.

Supplementary file1 (DOCX 34 KB)

Supplementary file2 (XLSX 24212 KB)

Supplementary file3 (XLSX 24049 KB)

Supplementary file4 (XLSX 24225 KB)

## Data Availability

All data supporting the findings of this study are available within the paper and its Supplementary Information files or from the corresponding author upon reasonable request.
