# Supplementary material for: Characterizing the immune response to myocardial infarction in pigs
Source: Basic Res Cardiol. 2024 Mar 15;119(3):453–79. doi: 10.1007/s00395-024-01036-2 (PMC11143055; doi:10.1007/s00395-024-01036-2)
Supplement: Supplementary file 1 — Supplementary file1 (DOCX 34 KB) [file 395_2024_1036_MOESM1_ESM.docx]

**Characterizing the immune response to myocardial infarction in pigs**

*Basic Research in Cardiology*

Florian Schnitter, Franziska Stangl, Elisabeth Noeske, Maya Bille, Anja Stadtmüller, Niklas Vogt, Florian Sicklinger, Florian Leuschner, Anna Frey, Laura Schreiber, Stefan Frantz, Niklas Beyersdorf, Gustavo Ramos, Nadine Gladow, Ulrich Hofmann

Corresponding author affiliation:

University Hospital Würzburg, Department of Internal Medicine I, Würzburg, Germany

Corresponding author e-mail:

schnitter_f@ukw.de

**Online Resource 1: Supplementary methods**

**Tab. 1** List of monoclonal primary antibodies used for flow cytometry (FC) and immunofluorescence microscopy (IFM)

| **Target**  **antigen** | **Antibody clone** | **Species host:target** | **Isotype/**  **subclass** | **Conjugate**  **(2nd antibody conjugate)** | **Source** | **Catalogue number** | **Final dilution** | **In**  **panel(s)** |
| --- | --- | --- | --- | --- | --- | --- | --- | --- |
| CD3ε | BB23-8E6-8C8 | mouse:pig | IgG2a | FITC  PE-Cy7 | BD | 559582  561477 | 1:200  1:200 | 4  2, 3 |
| CD4α | 74-12-4 | mouse:pig | IgG2b | PE | BD | 559586 | 1:200 | 2 |
| CD8α | 76-2-11 | mouse:pig | IgG2a | APC  FITC | Thermo Fisher | MA5-28712  MA5-28714 | 1:200  1:200 | 2  3 |
| CD8β | PPT23 | mouse:pig | IgG1 | DyLight 405 | Biorad | MCA5954GA* | 1:200 | 3 |
| CD14 | TÜK4 | mouse:human | IgG2a | Pacific Blue | Biorad | MCA1568PB | 1:25 | 1 |
| CD21^a^ | BB6-11C9.6 | mouse:pig | IgG1 | Alexa Fluor 700  FITC | Novus Biologicals | NBP1-28245AF700  NBP1-28246 | 1:200  1:200 | 3  4 |
| CD27 | B30C7 | mouse:pig | IgG1 | APC | Biorad | MCA5973F | 1:5 | 3 |
| CD31 | LCI-4 | mouse:pig | IgG1 | APC  FITC (PE) | Biorad | MCA1746APC  MCA1746F | 1:500  1:200 | 6  5 |
| CD45 | K252.1E4 | mouse:pig | IgG1 | Alexa Fluor 647  FITC | Biorad | MCA1222A647 MCA1222F | 1:100  1:50 | 5  1, 2 |
| CD79α | HM57 | mouse:human | IgG1 | Alexa Fluor 700 | Biorad | MCA2538A700 | 1:10 | 2 |
| CD117 | 2B8 | rat:mouse | IgG2b | PE-Cy7 | Thermo Fisher | 25-1171-82 | 1:50 | 4 |
| CD163 | 2A10/11 | mouse:pig | IgG1 | PE | Biorad | MCA2311GA* | 1:100 | 1 |
| CD172α | 74-22-15A | mouse:pig | IgG2b | APC-Cy7  PE  none (APC) | BD | 553640*  561499  553640 | 1:200  1:200  1:200 | 3  4, 6  1 |
| CD203α | PM18-7 | mouse:pig | IgG1 | FITC  none (PE-Cy7) | Biorad | MCA1973F  MCA1973GA | 1:100  1:50 | 7  1 |
| unknown | 6D10 | mouse:pig | IgG2a | none (Alexa Fluor 546) | Biorad | MCA2599GA | 1:100 | 7 |
| α-SMA | 1A4 | mouse:human | IgG2a | none (APC) | Biorad | MCA5781GA | 1:100 | 8 |
| CXCL8 | W12 | mouse:pig | IgG1 | none (APC) | Antibodies-online.com | ABIN6992650 | 1:50 | 7 |
| Foxp3 | FJK-16s | rat:mouse | IgG2a | PE-Cy5 | Thermo Fisher | 15-5773-82 | 1:50 | 2, 3 |
| Ki67 | B56 | mouse:human | IgG1 | Brilliant Violet 605 | BD | 567122 | 1:100 | 3, 4 |
| SWC8 | MIL3 | mouse:pig | IgM | none (APC-eFluor 780) | Thermo Fisher | MA5-28504 | 1:25 | 1 |
| Vimentin | RV202 | mouse:human | IgG1 | PE | BD | 562337 | 1:100 | 8 |

* originally unlabeled antibody, self-conjugated with the respective fluorochrome using a Lightning-Link kit (Abcam, Cambridge, UK) per manufacturer's instructions

Antibody panels: 1. myeloid cell panel (FC, heart), 2. lymphoid cell panel (FC, heart), 3. lymphoid cell panel (FC, lymph node), 4. stem/progenitor cell panel (FC, spleen), 5. pan-leukocyte panel (IFM, heart), 6. pan-myeloid cell panel (IFM, heart), 7. CXCL8 panel (IFM, heart), 8. (Myo)fibroblast panel (IFM, heart)

**Tab. 2** List of secondary antibodies used for flow cytometry (FC) and immunofluorescence microscopy (IFM)

| **Target antigen (primary antibody)** | **Antibody clone** | **Species**  **host:target** | **Isotype/**  **subclass** | **Conjugate** | **Source** | **Catalogue number** | **Final dilution** | **In panel** |
| --- | --- | --- | --- | --- | --- | --- | --- | --- |
| IgG (anti-6D10) | polyclonal | goat:mouse | IgG | Alexa Fluor 546 | Thermo Fisher | A-11003 | 1:200 | 7 |
| IgG1 (anti-CXCL8)  (anti-CD203α) | M1-14D12 | rat:mouse | IgG | APC  PE-Cy7 | Thermo Fisher | 17-4015-82  25-4015-82 | 1:200  1:200 | 7  1 |
| IgG2a (anti-α-SMA) | m2a-15F8 | rat:mouse | IgG1 | APC | Thermo Fisher | 17-4210-82 | 1:200 | 8 |
| IgG2b (anti-CD172α) | RMG2b-1 | rat:mouse | IgG1 | APC | BioLegend | 406712 | 1:200 | 1 |
| IgM (anti-SWC8) | II/41 | rat:mouse | IgG2a | APC-eFluor 780 | Thermo Fisher | 47-5790-82 | 1:200 | 1 |
| FITC (anti-CD31) | NAWESLEE | mouse:n/a | IgG2a | PE | Thermo Fisher | 12-7691-82 | 1:200 | 5 |

Antibody panels: 1. myeloid cell panel (FC, heart), 5. pan-leukocyte panel (IFM, heart), 7. CXCL8 panel (IFM, heart), 8. (Myo)fibroblast panel (IFM, heart)
